# Supplementary material for: In situ dual doping for constructing efficient CO2-to-methanol electrocatalysts
Source: Nat Commun. 2022 Apr 12;13:1965. doi: 10.1038/s41467-022-29698-3 (PMC9005706; doi:10.1038/s41467-022-29698-3)
Supplement: Supplementary file 1 — Supplementary Information [file 41467_2022_29698_MOESM1_ESM.pdf]

Supplementary Information for

In situ dual doping for constructing efficient CO_2-to-methanol electrocatalysts

Pengsong Li[1,2,3,4], Jiahui Bi[1,2,3,4], Jiyuan Liu[1,2,3,4], Qinggong Zhu[1,2,3,4,*], Chunjun Chen[1,2,3,4], Xiaofu Sun[1,2,3,4], Jianling Zhang[1,2,3,4], and Buxing Han[1,2,3,4,5,*]

[1] CAS Key Laboratory of Colloid, Interface and Chemical Thermodynamics, Institute of Chemistry, Chinese Academy of Sciences, Beijing 100190, P. R. China.

[2] Beijing National Laboratory for Molecular Sciences, Institute of Chemistry, Chinese Academy of Sciences, Beijing 100190, P. R. China.

[3] CAS Research/Education Center for Excellence in Molecular Sciences, Institute of Chemistry, Chinese Academy of Sciences, Beijing 100190, P. R. China.

[4] University of Chinese Academy of Sciences, Beijing 100049, P. R. China

[5] Shanghai Key Laboratory of Green Chemistry and Chemical Processes, School of Chemistry and Molecular Engineering, East China Normal University Shanghai 200062, P. R. China

[*]E-mail: B.X.H. (hanbx@iccas.ac.cn), Q.G.Z (qgzhu@iccas.ac.cn)

Contents

1. Supplementary Figures....4
Supplementary Fig. 1....4
Supplementary Fig. 2....5
Supplementary Fig. 3....6
Supplementary Fig. 4....7
Supplementary Fig. 5....8
Supplementary Fig. 6....9
Supplementary Fig. 7....10
Supplementary Fig. 8....11
Supplementary Fig. 9....12
Supplementary Fig. 10....13
Supplementary Fig. 11....14
Supplementary Fig. 12....15
Supplementary Fig. 13....16
Supplementary Fig. 14....17
Supplementary Fig. 15....18
Supplementary Fig. 16....19
Supplementary Fig. 17....20
Supplementary Fig. 18....21
Supplementary Fig. 19....22
Supplementary Fig. 20....23
Supplementary Fig. 21....24
Supplementary Fig. 22....25
Supplementary Fig. 23....26
Supplementary Fig. 24....27
Supplementary Fig. 25....28
Supplementary Fig. 26....29
Supplementary Fig. 27....30
Supplementary Fig. 28....31
Supplementary Fig. 29....32
Supplementary Fig. 30....33
Supplementary Fig. 31....34
Supplementary Fig. 32....35
Supplementary Fig. 33....36
Supplementary Fig. 34....37
Supplementary Fig. 35....38
Supplementary Fig. 36....39
Supplementary Fig. 37....40
Supplementary Fig. 38....41
2.Supplementary Table....42
Supplementary Table 1....42
Supplementary Table 2....44

Supplementary Table 3 45
Supplementary Table 4 47
3.References 50

1. Supplementary Figures

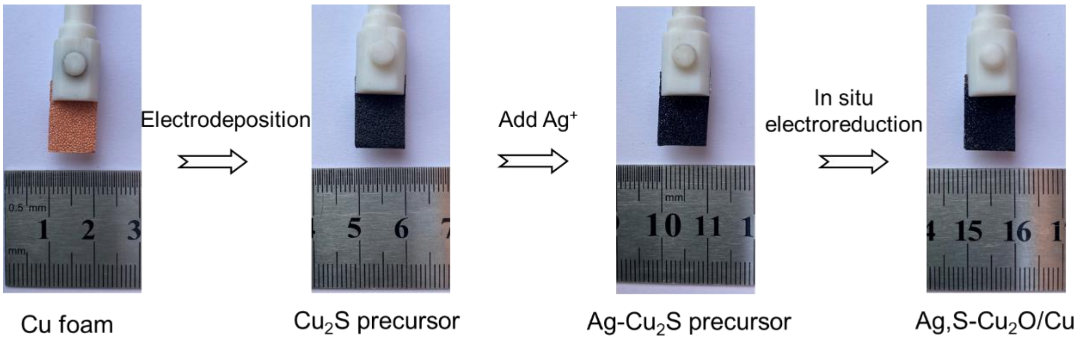

Supplementary Fig. 1. Optical images of Cu foam substrate, Cu_2S precursor, Ag-Cu_2S precursor and Ag,S-Cu_2O/Cu.

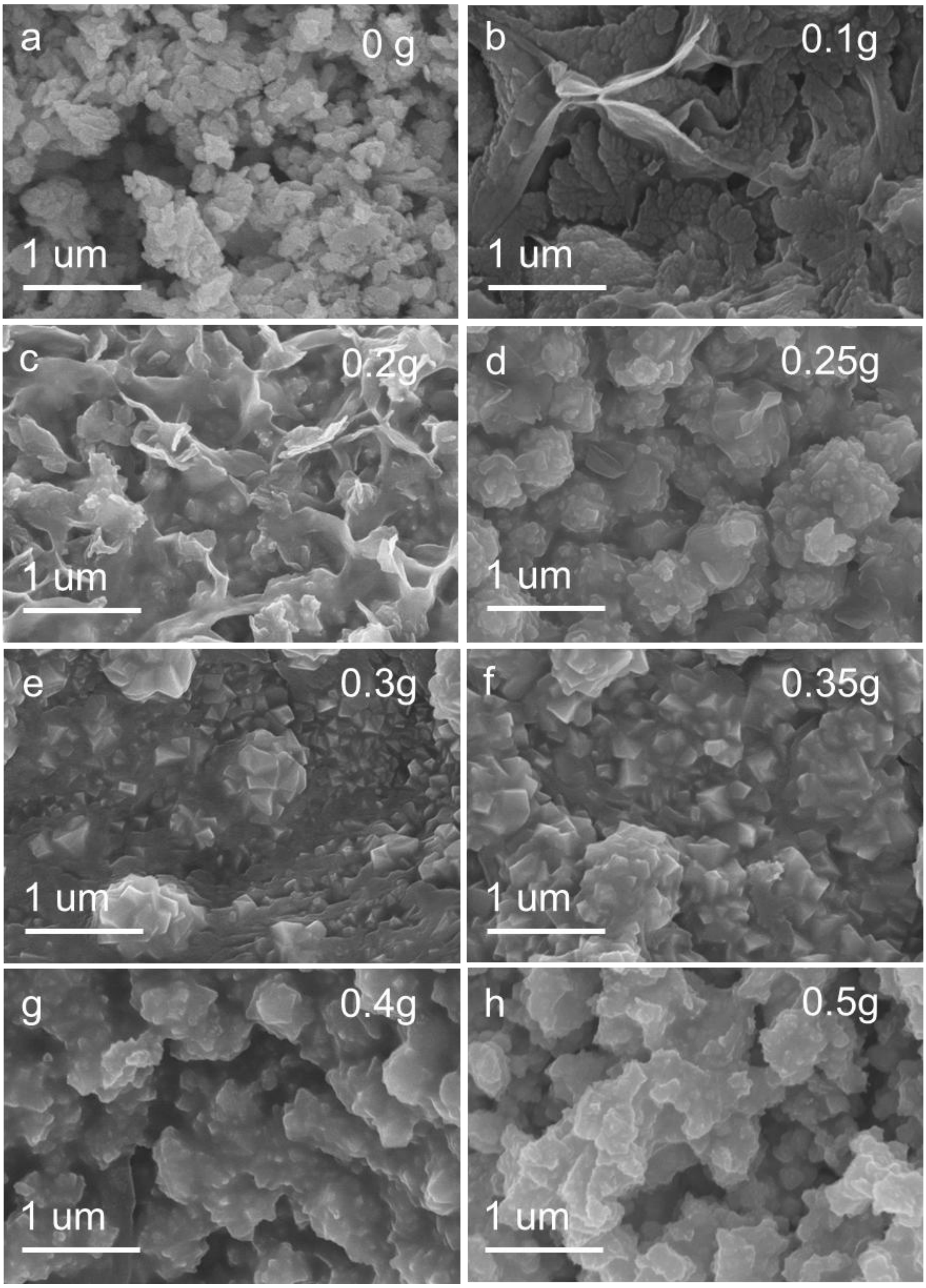

Supplementary Fig. 2. SEM images of Cu_2S precursor formed on Cu foam substrate with different CTAB amounts (0 g, 0.1g, 0.2g, 0.25g, 0.3g, 0.35g, 0.4g, 0.5g (a-h)).

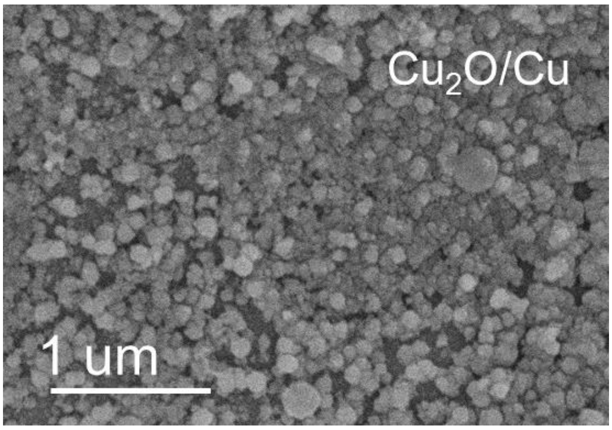

Supplementary Fig. 3. SEM image of Cu_2O on Cu foam substrate at electroreduction time of 30 min.

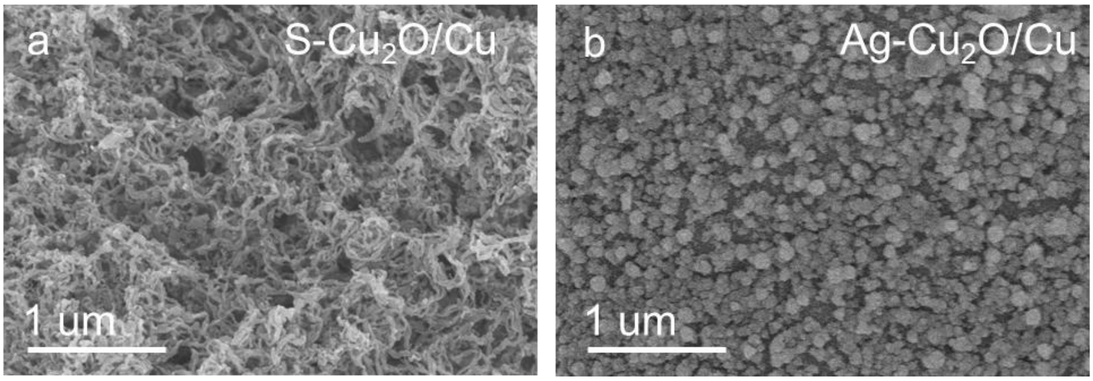

Supplementary Fig. 4. SEM images of S-Cu_2O/Cu (a) and Ag-Cu_2O/Cu (b) on Cu foam substrate at electroreduction time of 30 min.

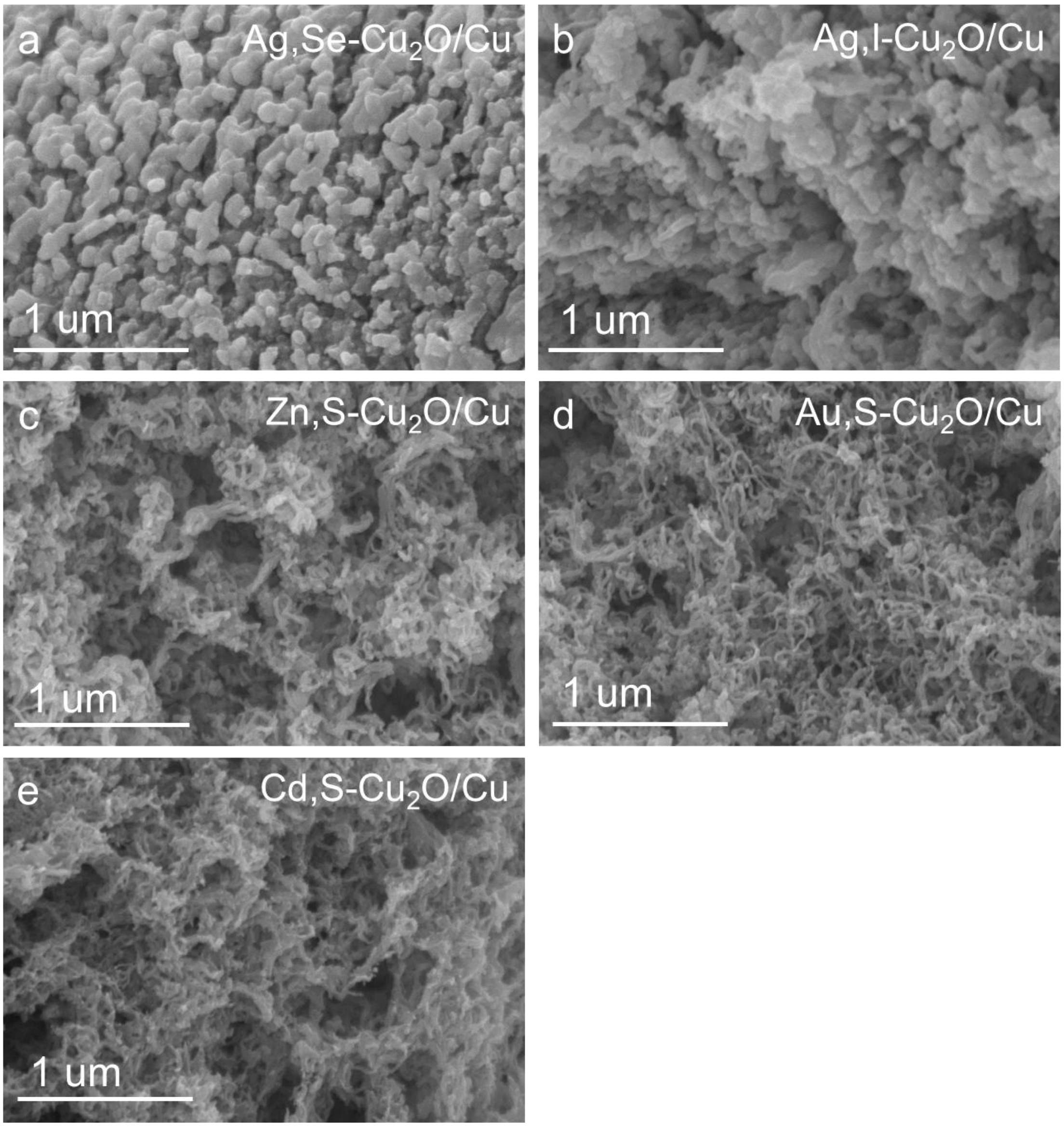

Supplementary Fig. 5. SEM images of (a) Ag,Se-Cu_2O/Cu, (b) Ag,I-Cu_2O/Cu, (c) Zn,S-Cu_2O/Cu, (d) Au,S-Cu_2O/Cu and (e) Cd,S-Cu_2O/Cu on Cu foam substrate at electroreduction time of 30 min.

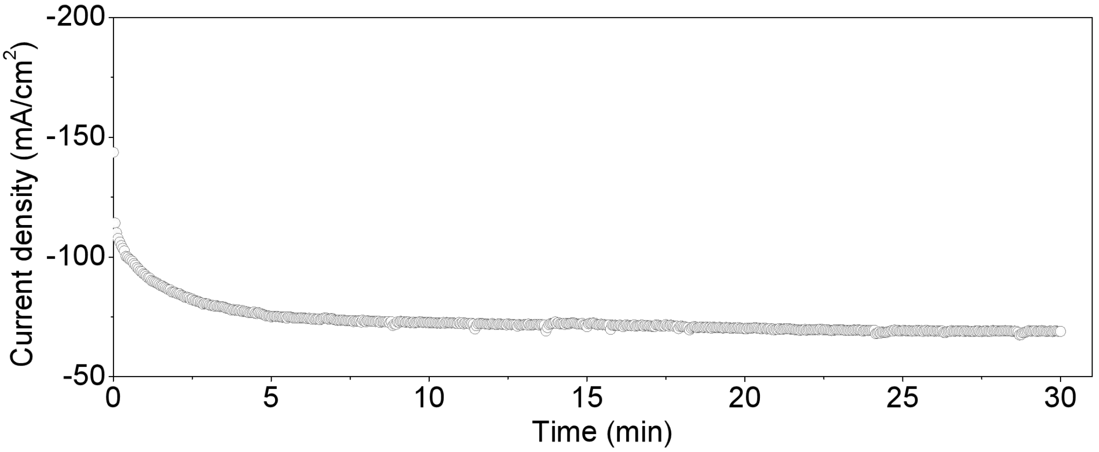

Supplementary Fig. 6. Current density curve of the in situ conversion of Ag-Cu_2S precursor to dual doping Ag,S-Cu_2O/Cu porous nanonetwork on the Cu foam substrate.

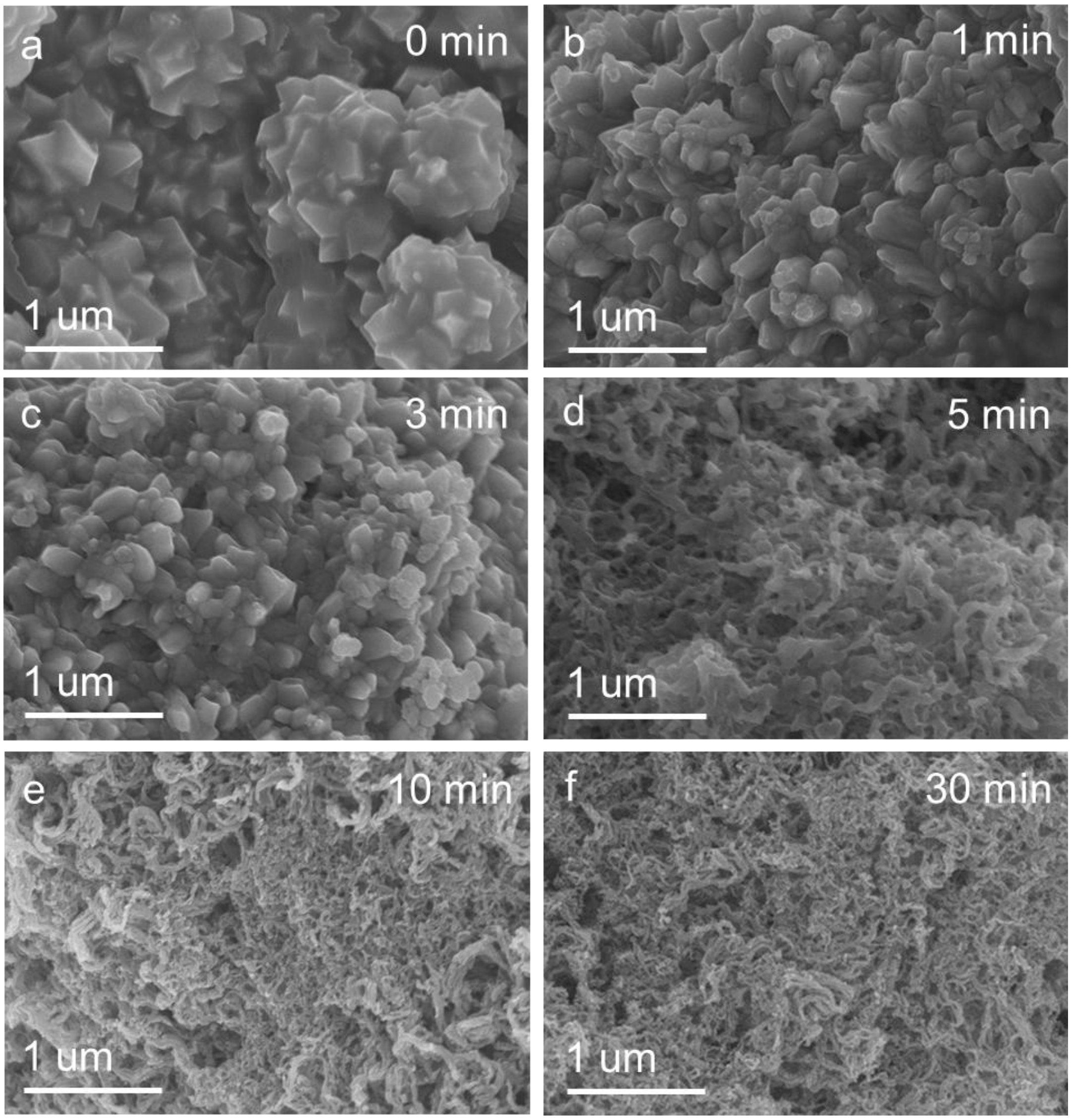

Supplementary Fig. 7. SEM images of Ag,S-Cu_2O/Cu formed at different electroreduction time (0min, 1min, 3min, 5min,10min, 30 min (a-f)).

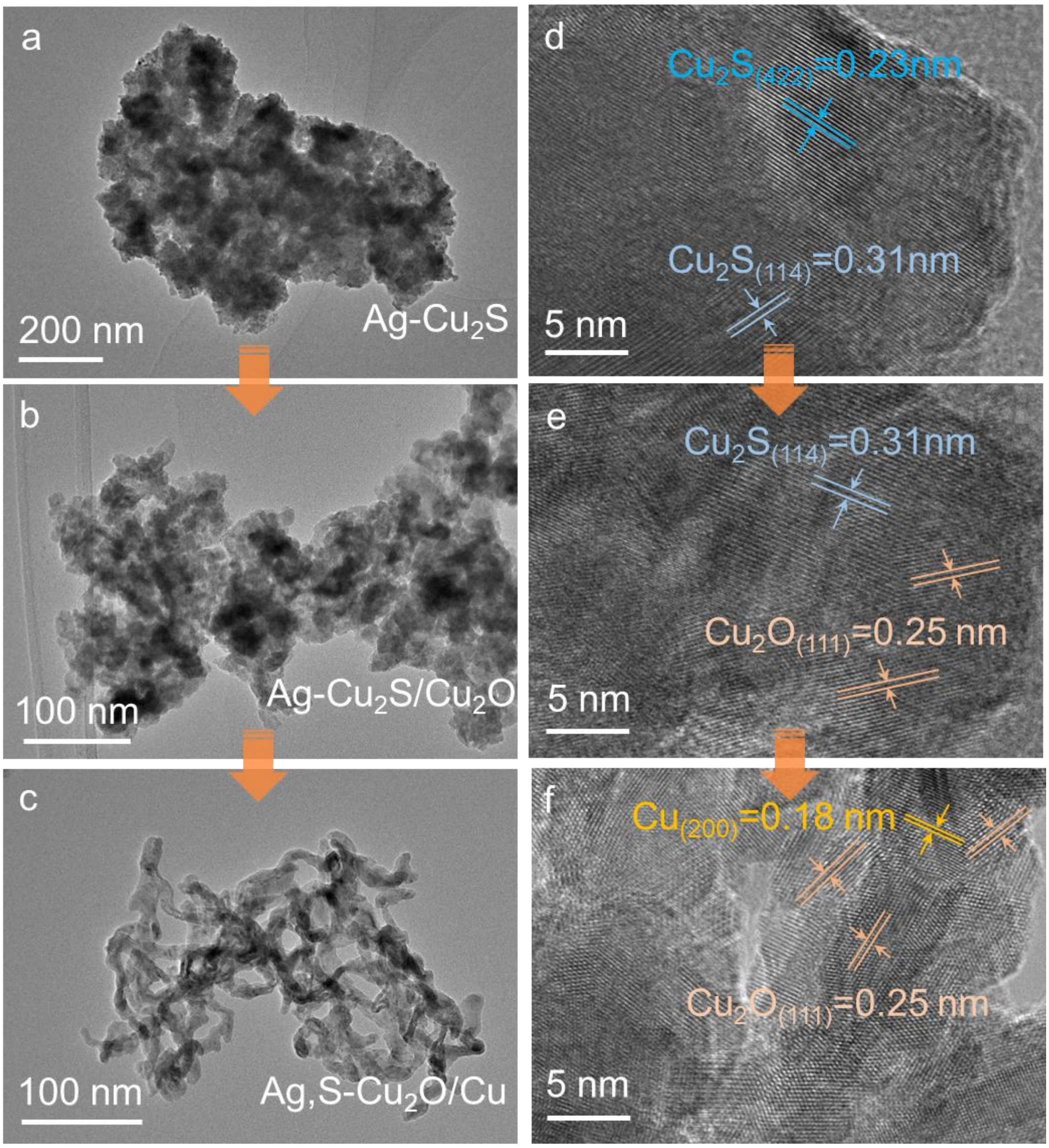

Supplementary Fig. 8. (a-c) TEM and (d-f) HRTEM images of Ag-Cu_2S precursor to Ag,S-Cu_2O/Cu at reduction time of 0 min, 3 min and 30 min.

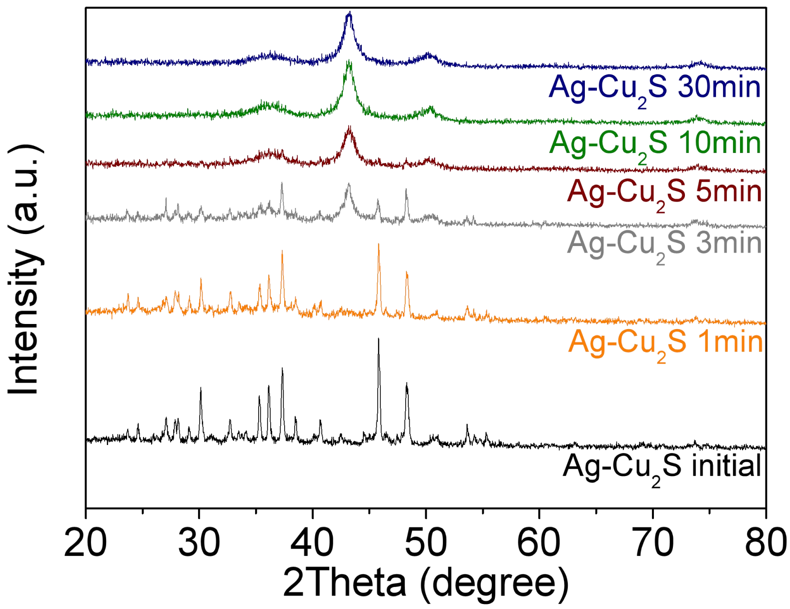

Supplementary Fig. 9. XRD patterns of Ag,S-Cu_2O/Cu formed at different electroreduction time.

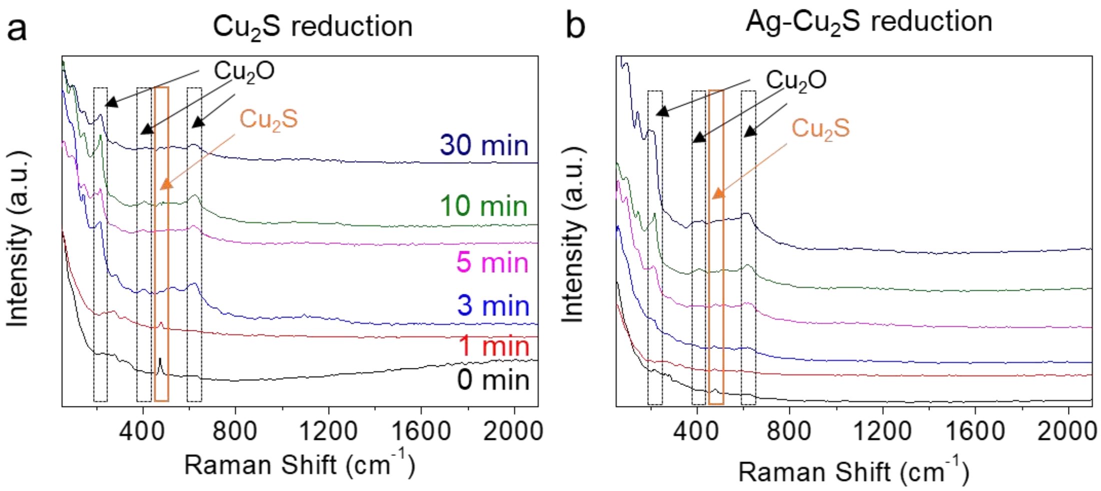

Supplementary Fig. 10. Raman spectra of Cu_2S and Ag-Cu_2S at different electroreduction time.

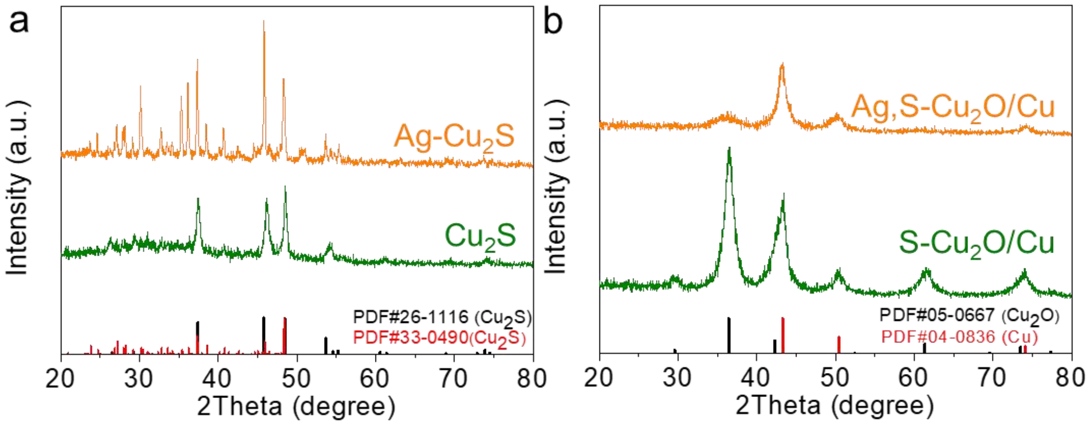

Supplementary Fig. 11. XRD patterns of (a) Cu_2S, Ag-Cu_2S precursors and (b) S-Cu_2O/Cu, Ag,S-Cu_2O/Cu formed at electroreduction time of 30 min.

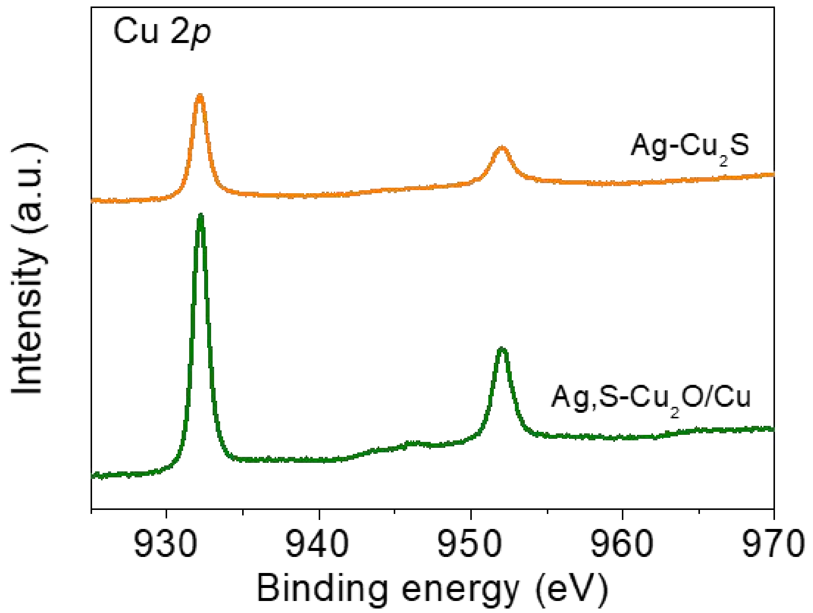

Supplementary Fig. 12. Quasi-in situ XPS spectra of Cu 2*p* in the Ag-Cu_2S precursor and Ag,S-Cu_2O/Cu with electroreduction time of 30 min.

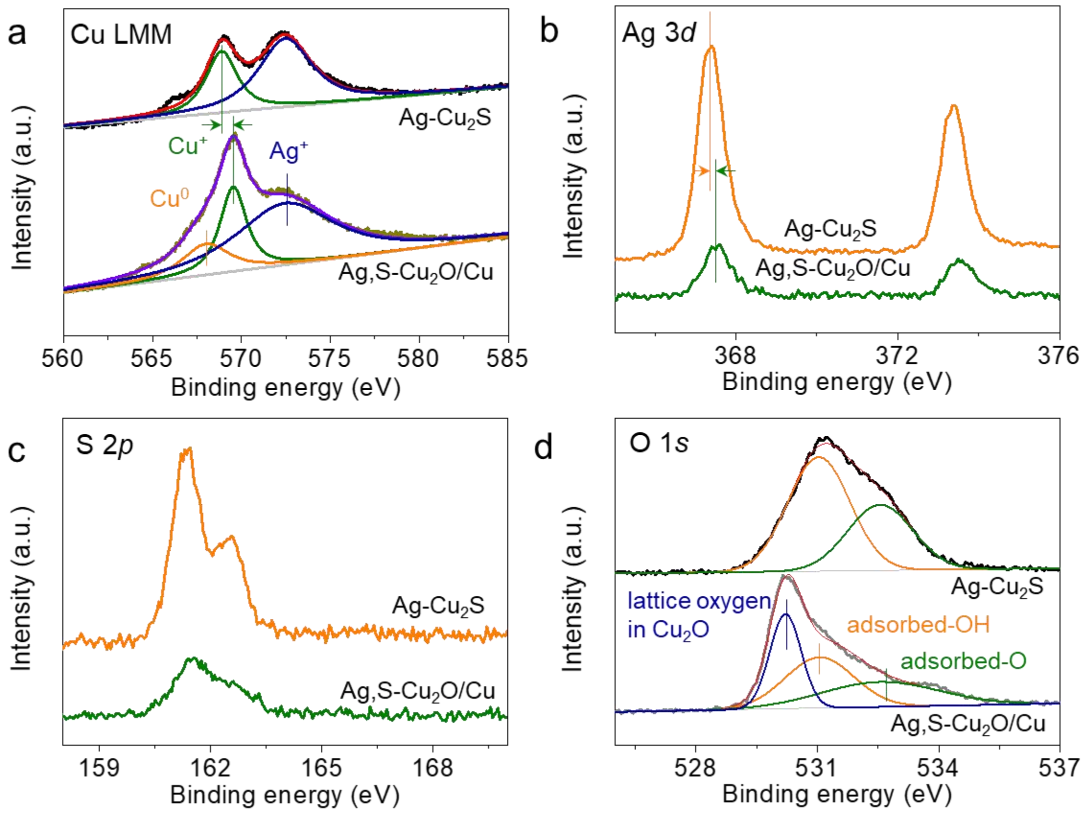

Supplementary Fig. 13. Quasi-in situ XPS spectra of Cu LMM (a), Ag 3*d* (b) S 2*p* (c) and (d) O 1*s* in the Ag-Cu_2S precursor and Ag,S-Cu_2O/Cu with electroreduction time of 30 min.

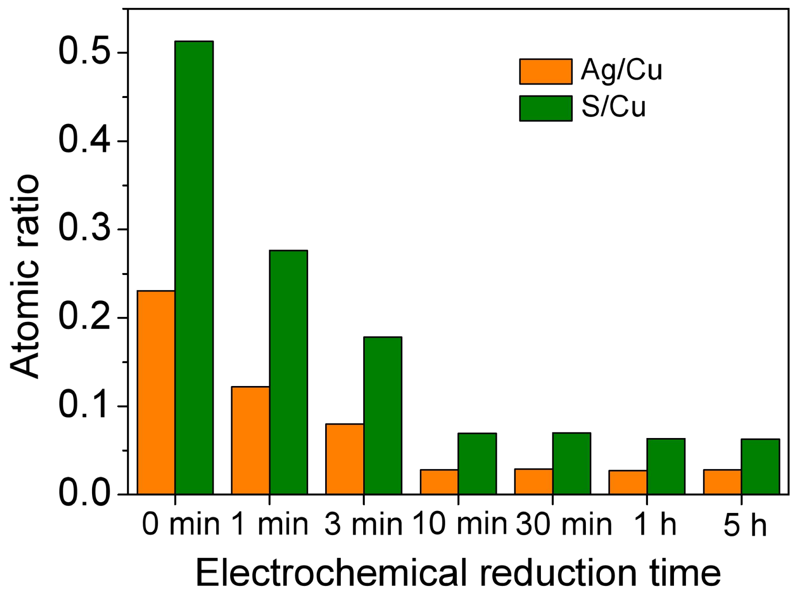

Supplementary Fig. 14. Amount evolution of Ag and S in the Ag,S-Cu_2O/Cu electrode with different electroreduction time (0 min, 1 min, 3 min, 10 min, 30 min, 1 h, 5 h). It was calculated from the corresponding peak area ratio in the XPS spectra.

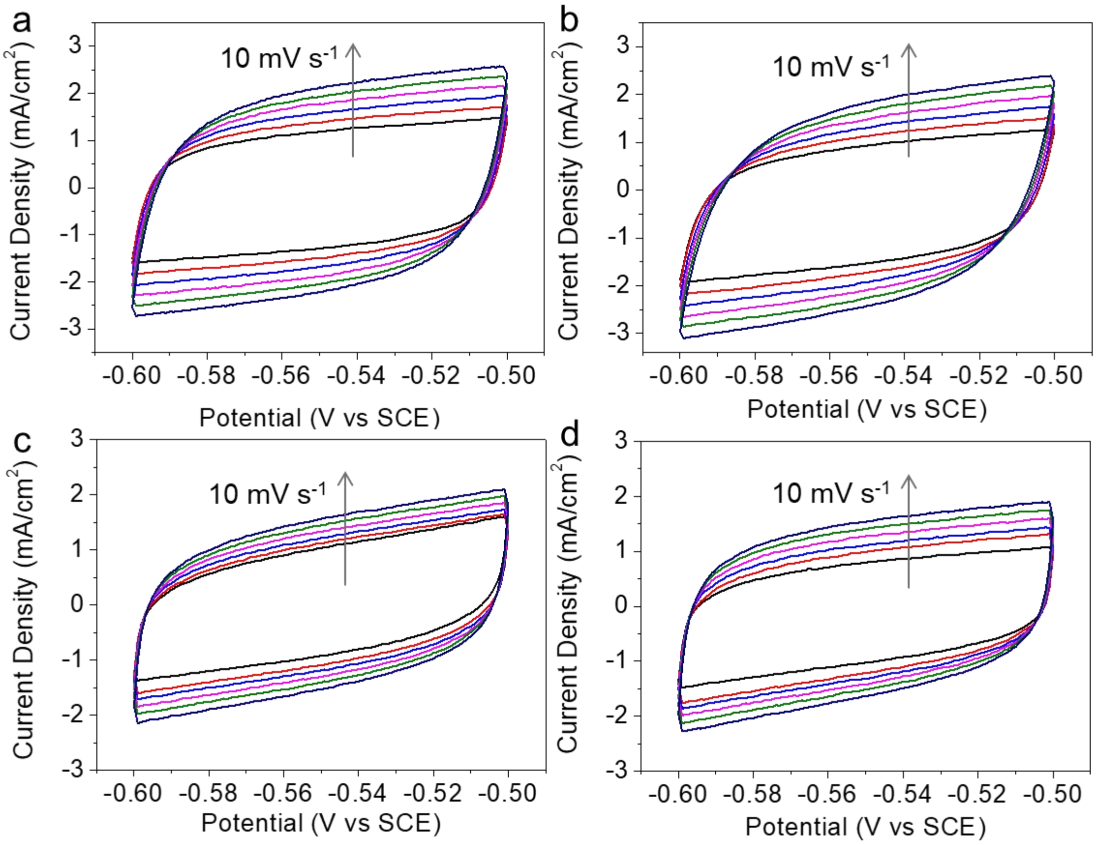

Supplementary Fig. 15. Electric double layer capacitance (C_{dl}) measurements at the non-Faradaic region (from -0.5 to -0.6 V vs. SCE) with various scan rates (50 mV s^{-1}-100 mV s^{-1}) of (a) Ag,S-Cu_2O/Cu, (b) S-Cu_2O/Cu, (c) Ag-Cu_2O/Cu and (d) S-Cu_2O/Cu.

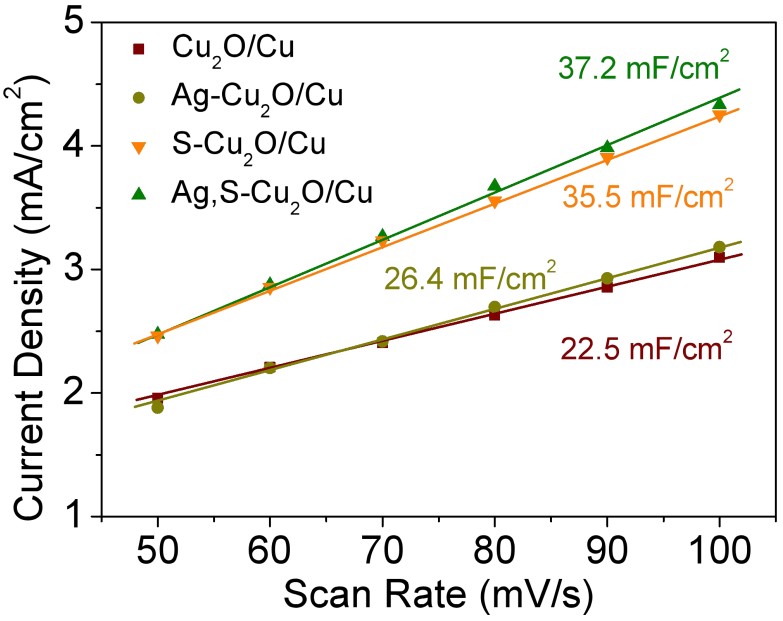

Supplementary Fig. 16. Charging current density differences plotted against scan rates.

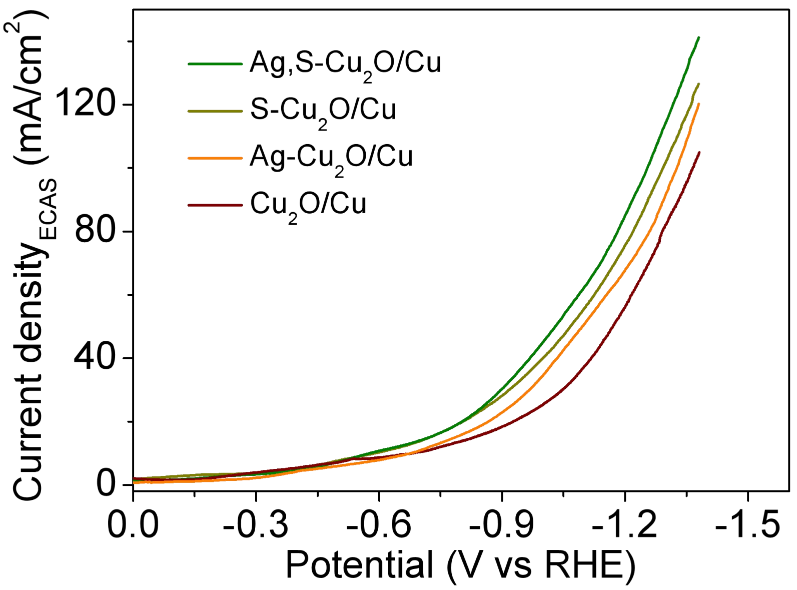

Supplementary Fig. 17. Linear sweep voltammetry curves of various catalysts in CO_2-saturated $BMImBF_4/H_2O$ electrolyte at the scan rate of 10 mV s^{-1}. The current density is normalized to ECSA.

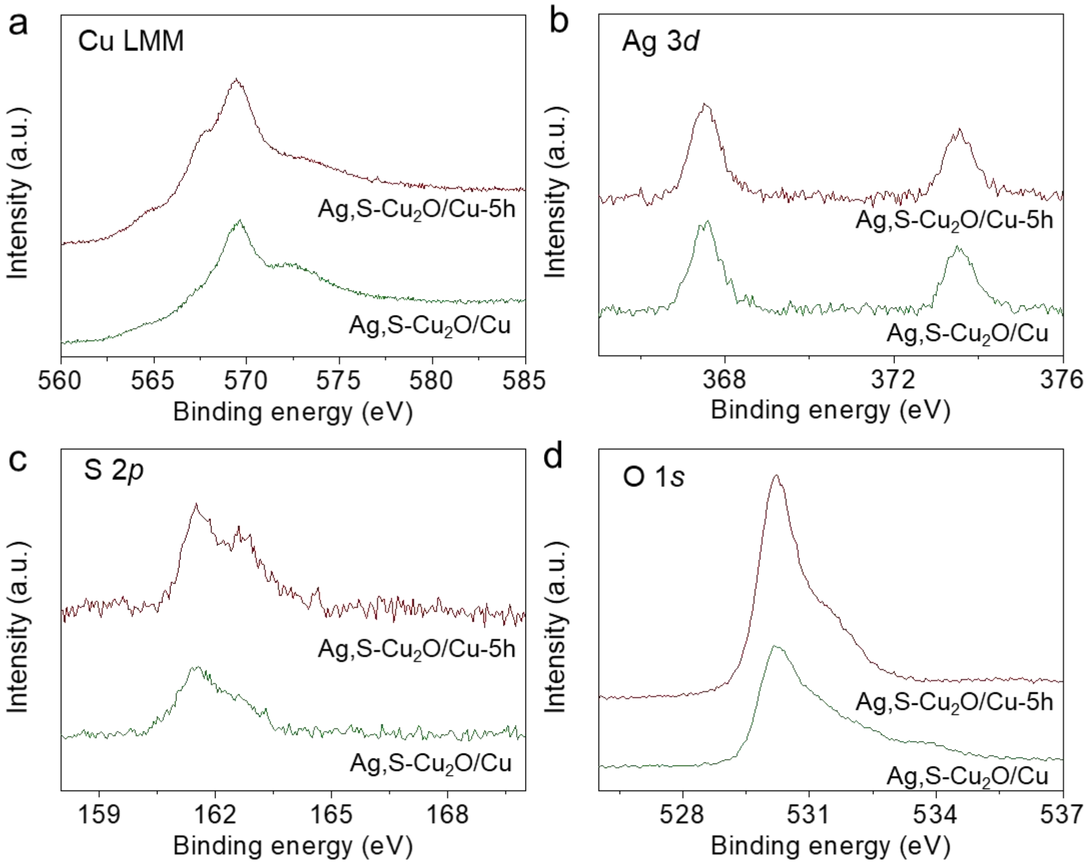

Supplementary Fig. 18. Quasi-in situ XPS spectra of Cu LMM (a), Ag 3*d* (b) S 2*p* (c) and (d) O 1*s* in the Ag,S-Cu_2O/Cu before and after 5 h CO_2 electrolysis.

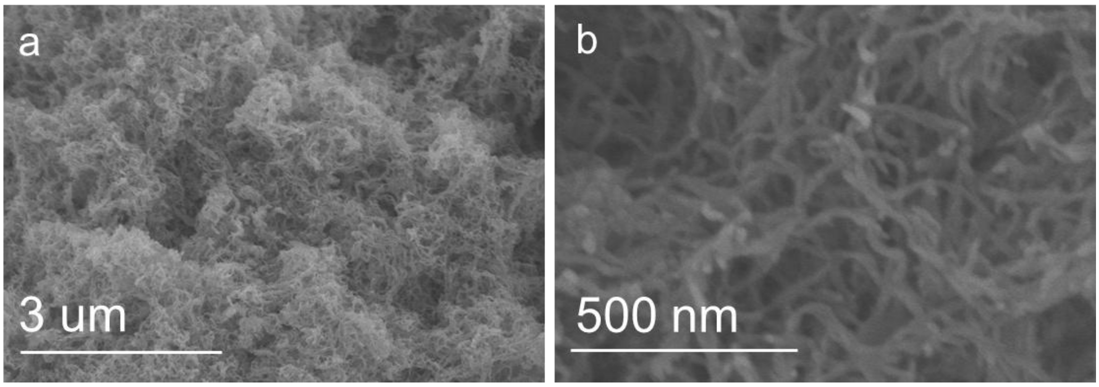

Supplementary Fig. 19. SEM images of the Ag,S-Cu_2O/Cu electrode after 24 h CO_2 electrolysis.

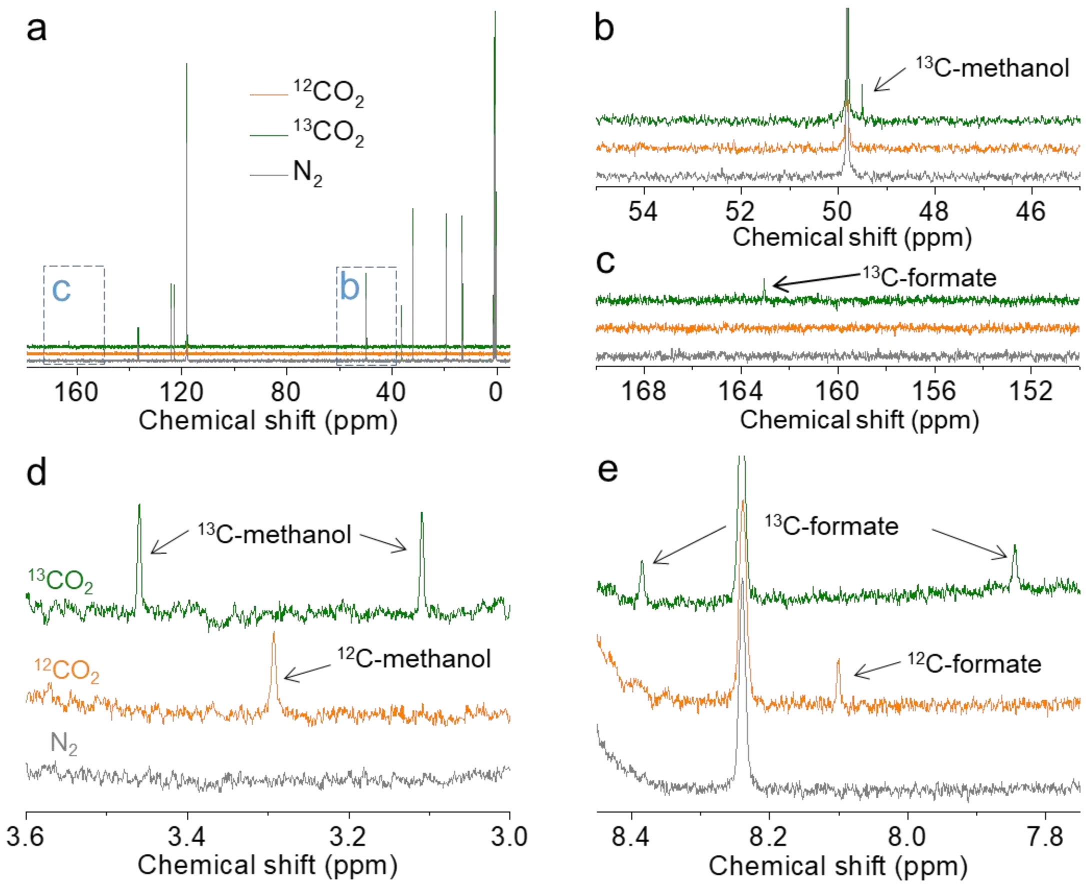

Supplementary Fig. 20. ^{13}C NMR (a, b, c) and ^{1}H NMR (d, e) spectra of the electrolyte solutions after electrolysis using N_2, $^{12}CO_2$ and $^{13}CO_2$ as the feedstock over Ag,S-Cu_2O/Cu electrode at an applied potential of -1.18 V vs. RHE in gas-saturated $BmimBF_4$/H_2O(with mole ratio of 1:3) solution.

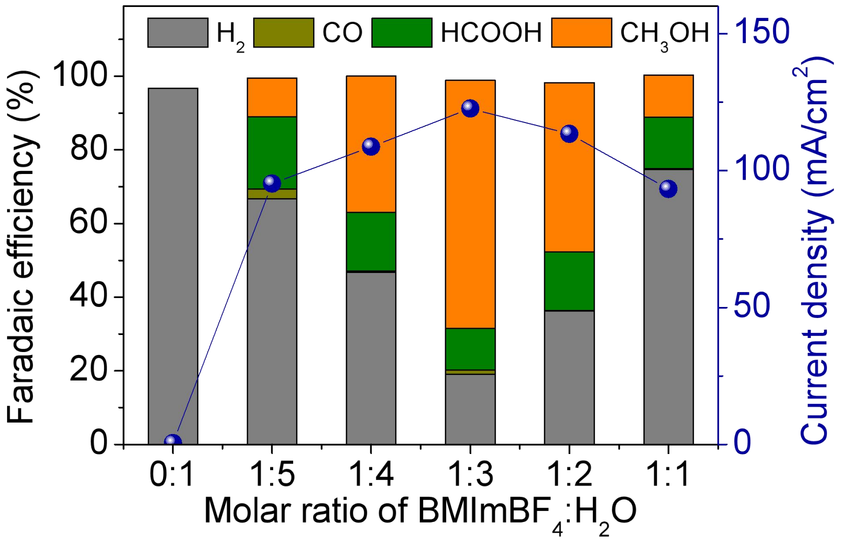

Supplementary Fig. 21. Product selectivity and current density for CO_2RR over Ag,S-Cu_2O/Cu in CO_2-saturated $BMImBF_4/H_2O$ electrolyte with different molar ratios at the potential of -1.18 V vs. RHE. It shows that the Ag,S-doped catalyst does not have the activity towards methanol with a very low current density of 0.51 mA cm^{-2} in the pure H_2O electrolyte. With increasing ionic liquid content in the electrolyte, the current density increased to 122.7 mA cm^{-2} (the molar ratio of $BMImBF_4/H_2O$ is 1:3) with the highest FE of CH_3OH and then decreased gradually. When the molar ratio exceeded 1:3, the motion of the ions was hindered and the rate of charge transfer was reduced on the electrode surface which was mainly caused by the high viscosity and electrostatic attraction between the anions and cations of the $BMImBF_4$.

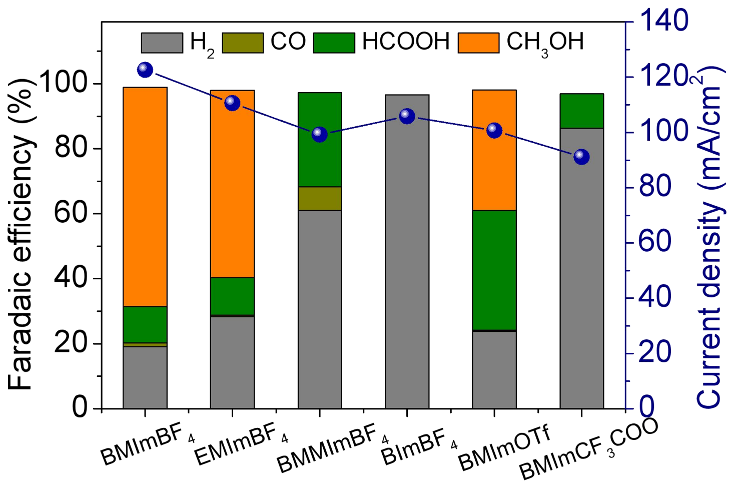

Supplementary Fig. 22. Influence of ionic liquids for the electrochemical CO_2 reduction of Ag,S-Cu_2O/Cu electrode (the molar ratio of IL/H_2O is 1:3). To understand the role of cations and anions in the IL, some other common ionic liquids with H_2O were also selected as electrolyte, including 1-butyl-2,3-dimethylimidazolium tetrafluoroborate ($BMMImBF_4$), 1-ethyl-3-ethylimidazolium tetrafluoroborate ($EMImBF_4$), 1-butylimidazolium tetrafluoroborate ($BImBF_4$), 1-butyl-3-methylimidazolium trifluoroacetate ($BMImCF_3COO$) and 1-butyl-3-methylimidazolium trifluoromethanesulfonate (BMImOTf).

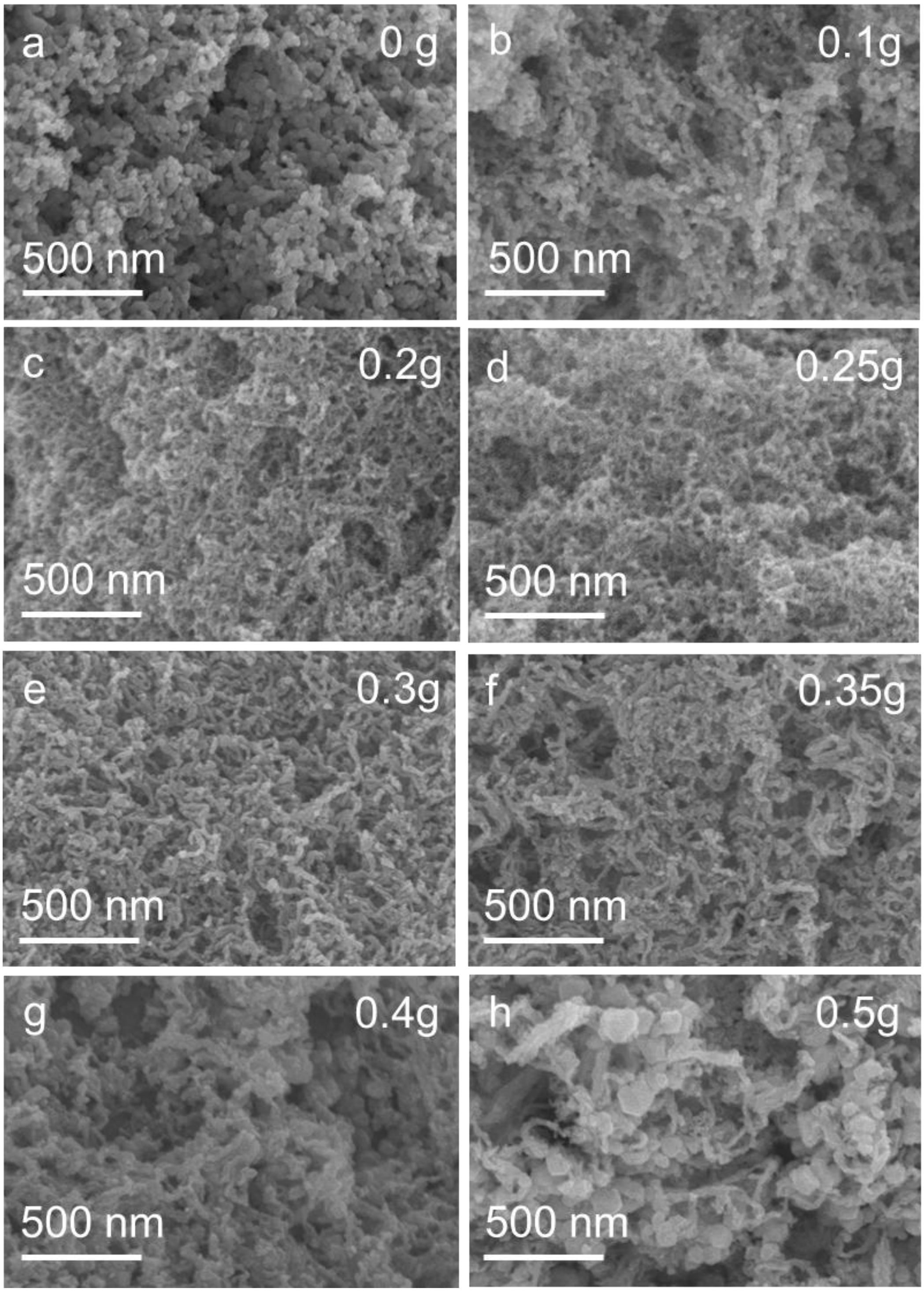

Supplementary Fig. 23. SEM images of Ag,S-Cu_2O/Cu formed at the electroreduction time of 30 min with different CTAB amounts (0 g, 0.1g, 0.2g, 0.25g, 0.3g, 0.35g, 0.4g, 0.5g (a-h)).

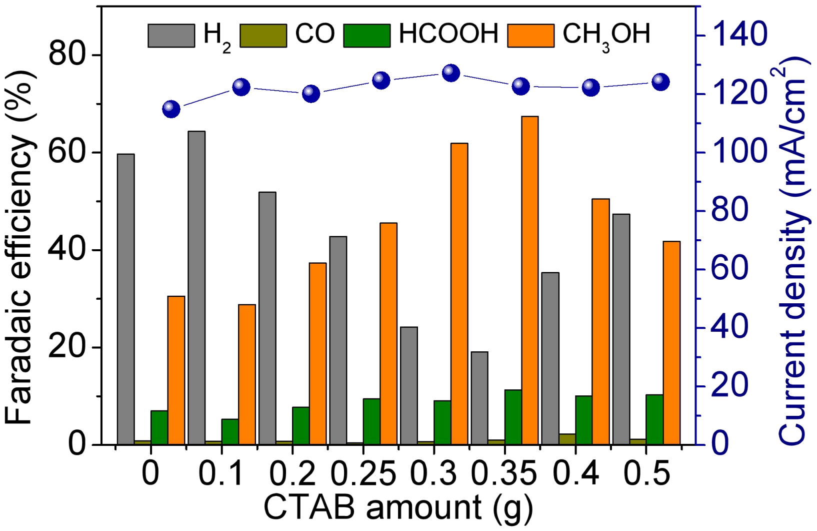

Supplementary Fig. 24. Influence of CTAB amount added when preparing Ag,S-Cu_2O/Cu electrode on the electrochemical CO_2 reduction.

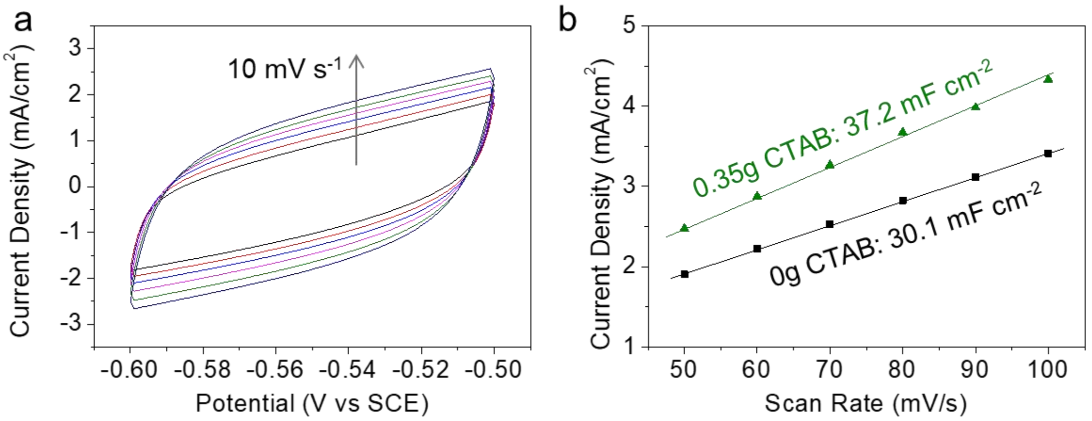

Supplementary Fig. 25. C_{dl} measurements at the non-Faradaic region (from -0.5 to -0.6 V vs. SCE) with various scan rates (50 mV s^{-1}-100 mV s^{-1}) of (a) Ag,S-Cu_2O/Cu with nanoparticle structure (0 g CTAB). (b) Charging current density differences plotted against scan rates(0 g CTAB and 0.35 g CTAB).

a

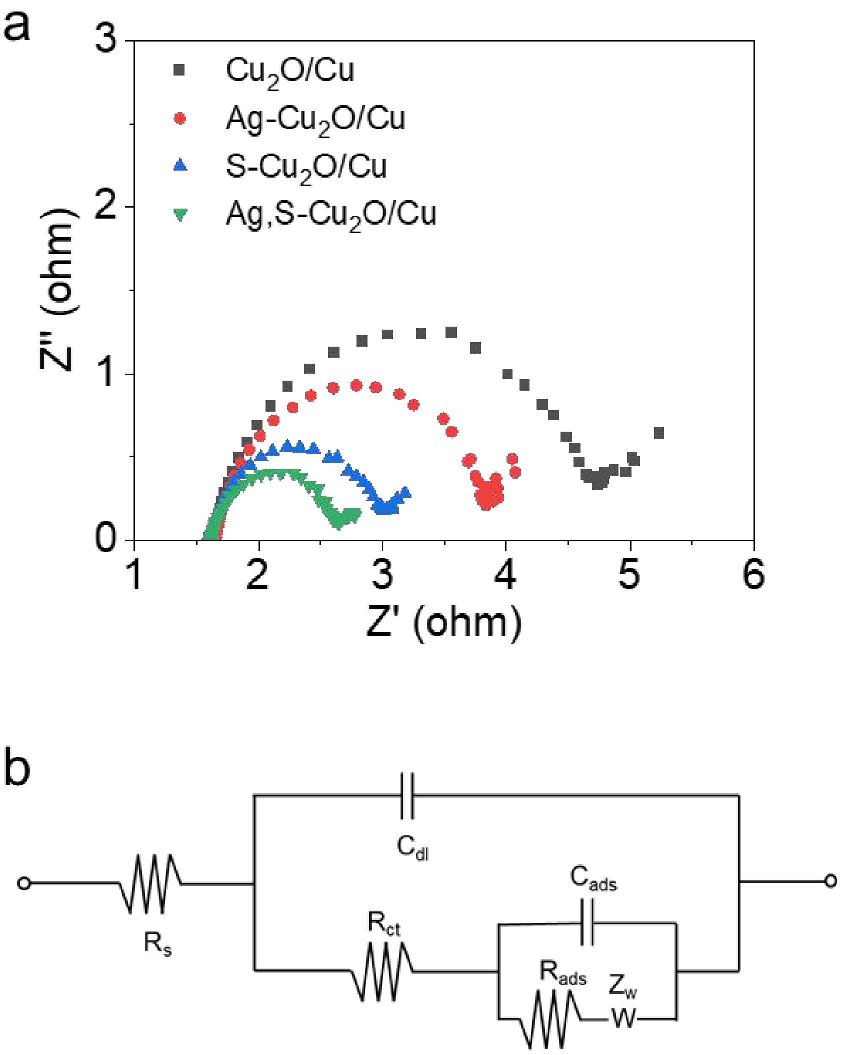

Supplementary Fig. 26. (a) EIS curves of Cu_2O/Cu, S-Cu_2O/Cu, Ag-Cu_2O/Cu and Ag,S-Cu_2O/Cu in CO_2-saturated $BMImBF_4/H_2O$ (mole ratio 1:3) electrolyte at the potential of -1.18 V vs. RHE. (b) Randles' equivalent circuit used for fitting the experimental impedance data. R_s, C_{dl}, R_{ct}, R_{ads}, C_{ads}, and Z_w stand for the solution resistance, double-layer capacitance, charge transfer resistance, surface adsorption resistance, surface adsorption capacitance, and Warburg-type impedance, respectively.

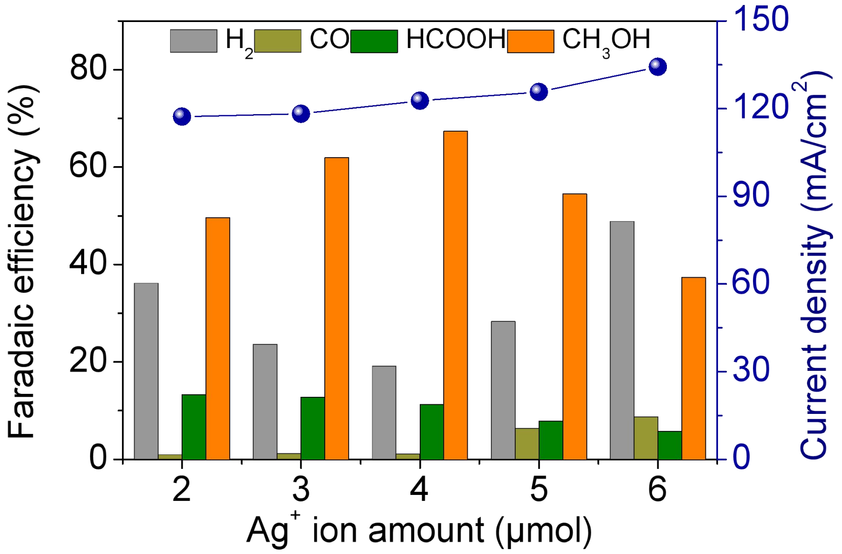

Supplementary Fig. 27. Electrochemical CO_2 reduction performance of Ag,S-Cu_2O/Cu electrode with different Ag^+ ion amounts.

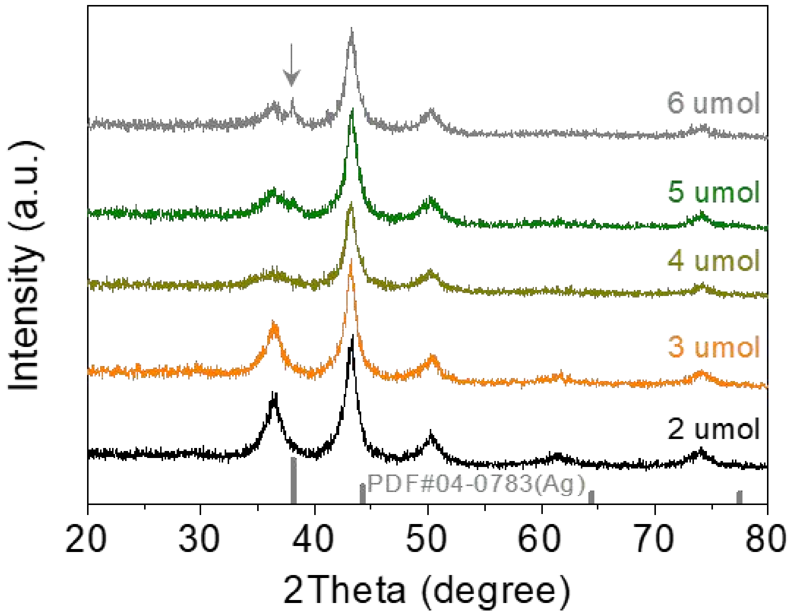

Supplementary Fig. 28. XRD patterns of Ag,S-Cu_2O/Cu electrode with different Ag^+ ion amounts.

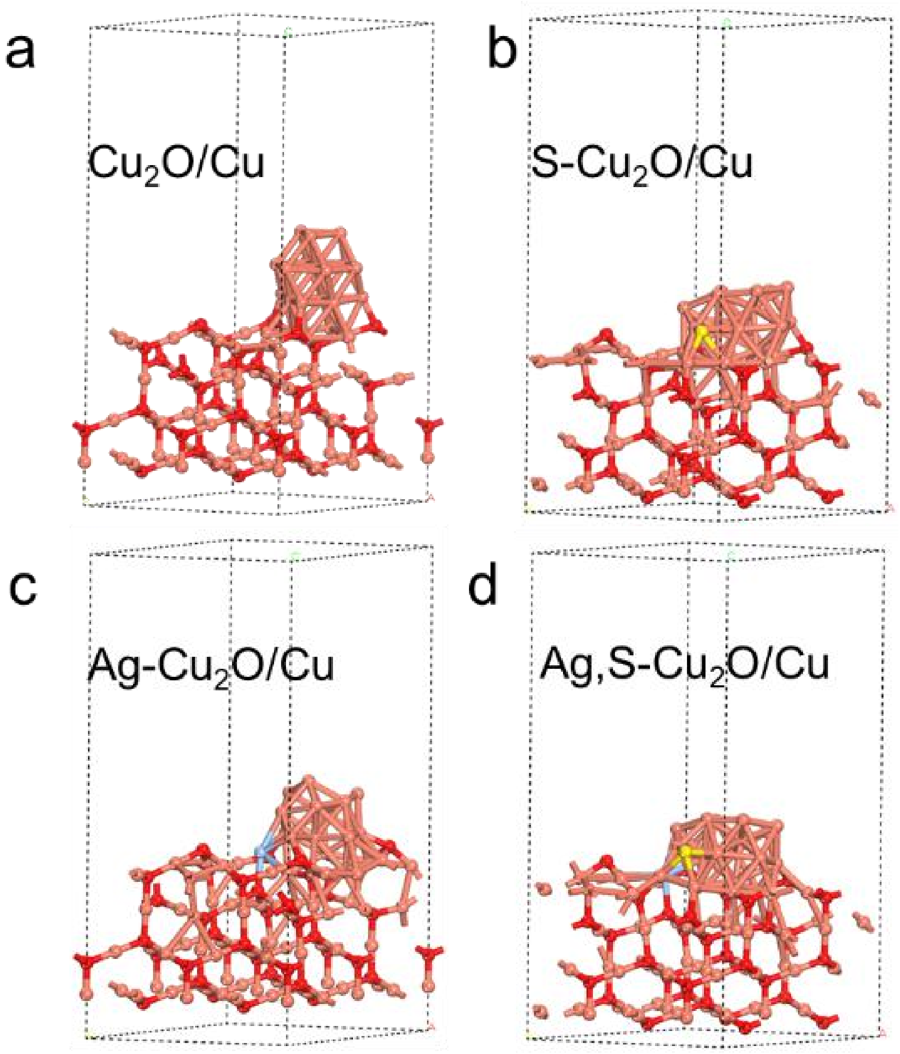

Supplementary Fig. 29. Schematic structures of (a) Cu_2O/Cu, (b) S-Cu_2O/Cu, (c) Ag-Cu_2O/Cu and (d) Ag,S-Cu_2O/Cu. The atoms in orange, light blue, grey, red, yellow and white represent Cu, Ag, C, O, S and H, respectively.

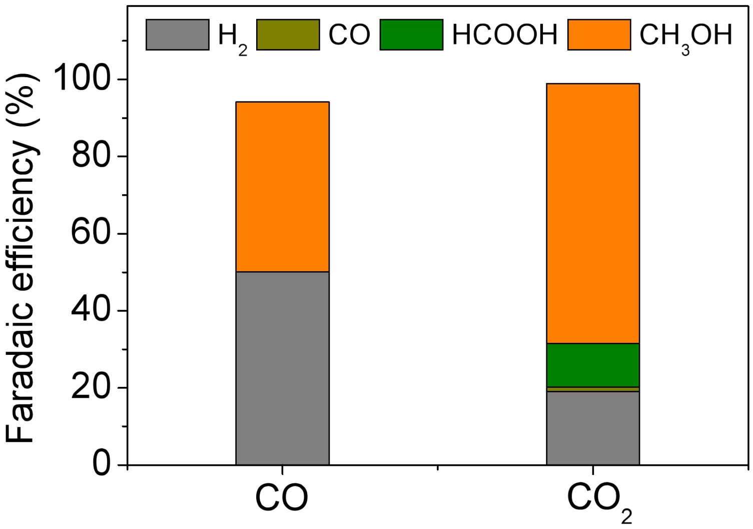

Supplementary Fig. 30. Electrochemical performance of Ag,S-Cu_2O/Cu using CO_2 or CO as the feeding gas at the potential of -1.18 V vs. RHE. For an Ag/Cu tandem system, the improving selectivity may be mainly originated from Ag that can efficiently generate CO for further reduction. However, the Ag and Cu in our catalyst do not constitute a tandem catalyst, as confirmed by our experimental findings (**Fig. 2**). The FE of methanol over Cu_2O/Cu, mono-doped Ag-Cu_2O/Cu and S-Cu_2O/Cu were 3.5%, 25.0% and 46.4%, respectively. It indicates that both Ag-Cu and S-Cu system can promote methanol production. When the Ag and S are doped simultaneously, the FE of methanol is maximized (67.4%), which should be attributed to the synergy of Ag and S, not just Ag. To further demonstrate this point, we design some control experiments. We performed the electrochemical test with CO as the feeding gas, assuming that enough CO was generating on the catalyst surface. It is obvious that the FE of methanol in the presence of CO is significantly lower than that of CO_2 as the feed gas (**Supplementary Fig. 30**). This suggests that sufficient CO is not a decisive factor for improving methanol selectivity over our Ag,S-Cu_2O/Cu catalyst. Based on the above results, we proposed that Ag and S can synergistically tune the electronic structure of nearby Cu active sites, making methanol production more accessible.

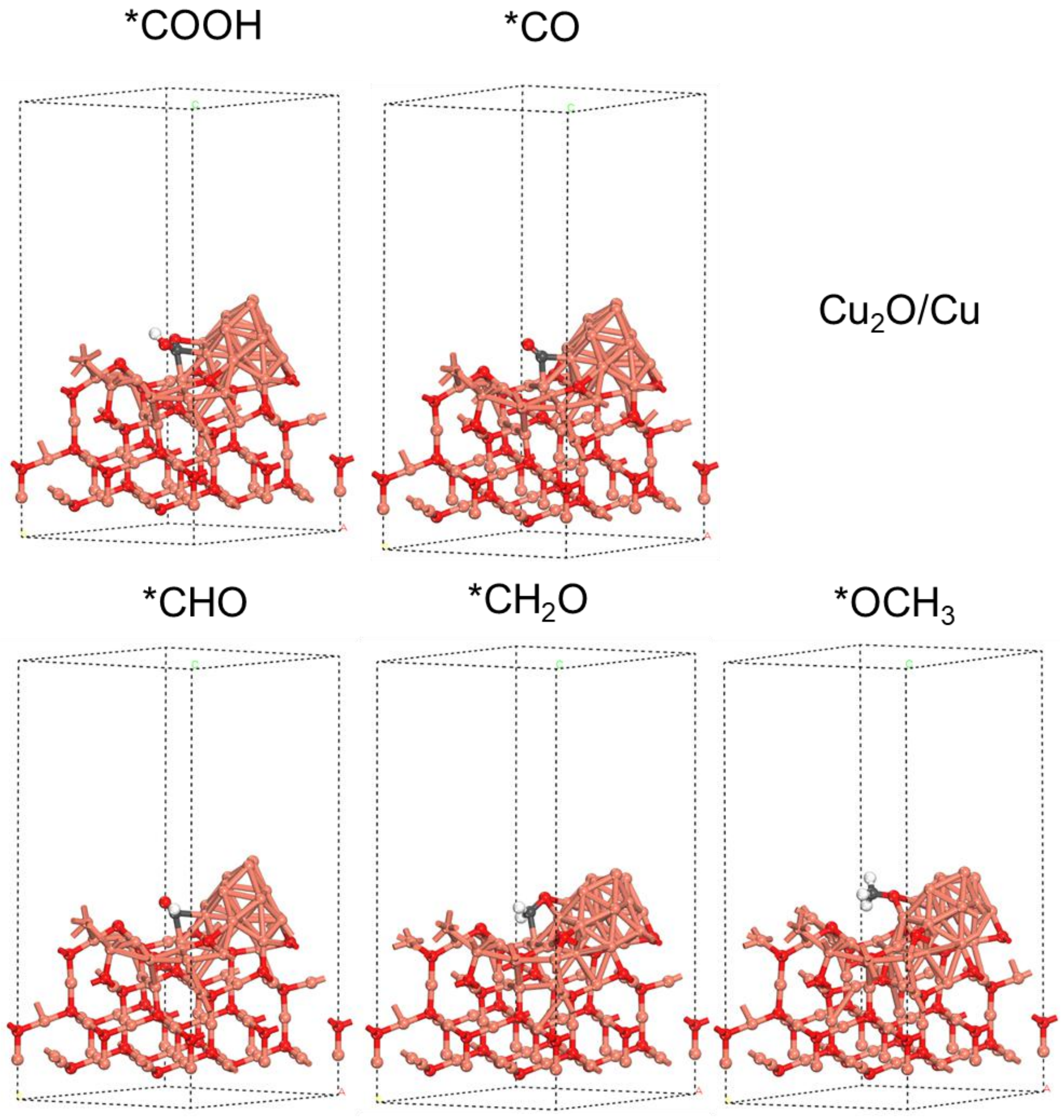

Supplementary Fig. 31. The optimized adsorption configurations of reaction intermediates on the Cu_2O/Cu structure. The atoms in orange, grey, red and white represent Cu, C, O and H, respectively.

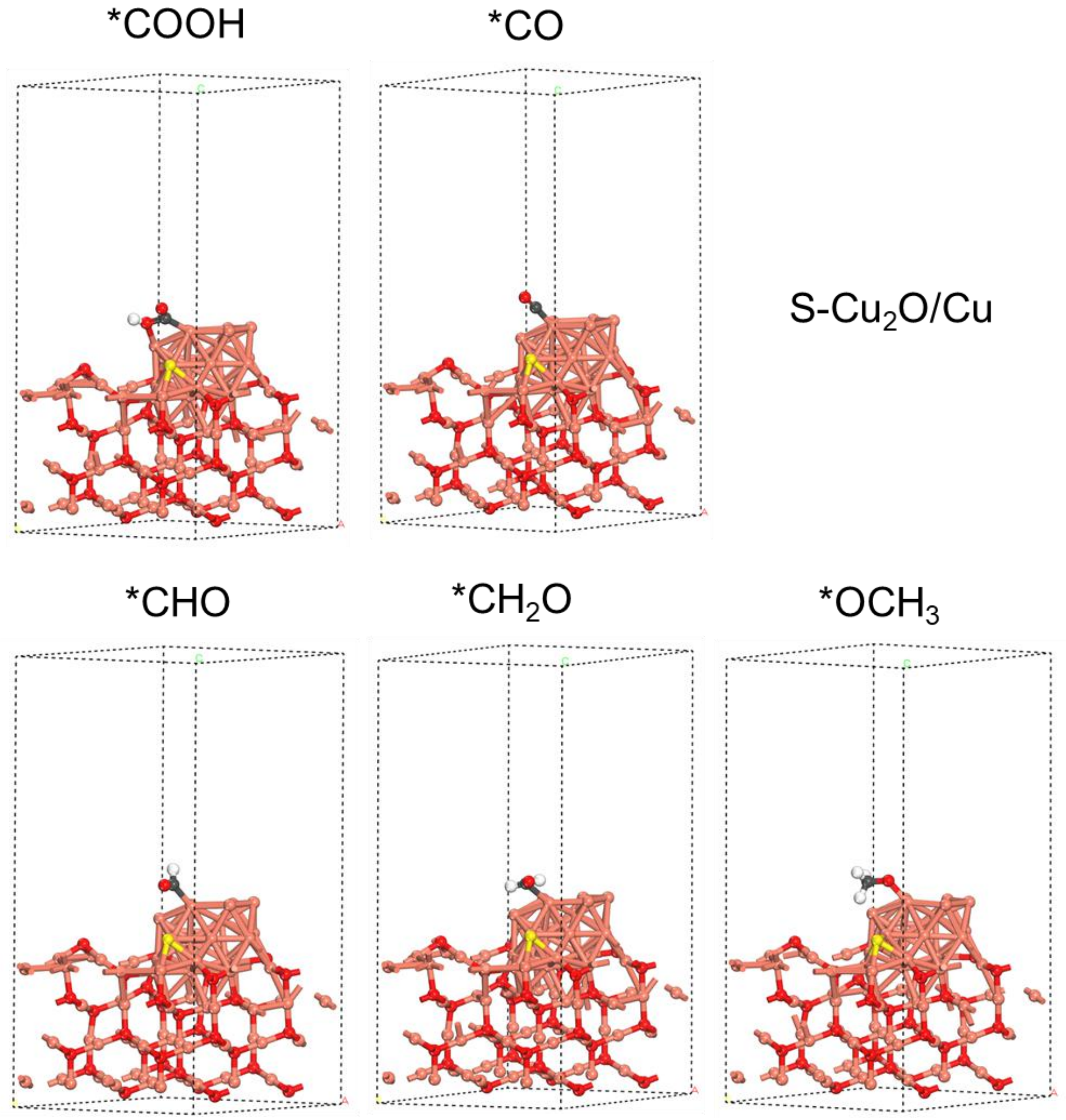

Supplementary Fig. 32. The optimized adsorption configurations of reaction intermediates on the S-Cu_2O/Cu structure. The atoms in orange, grey, red, yellow and white represent Cu, C, O, S and H, respectively.

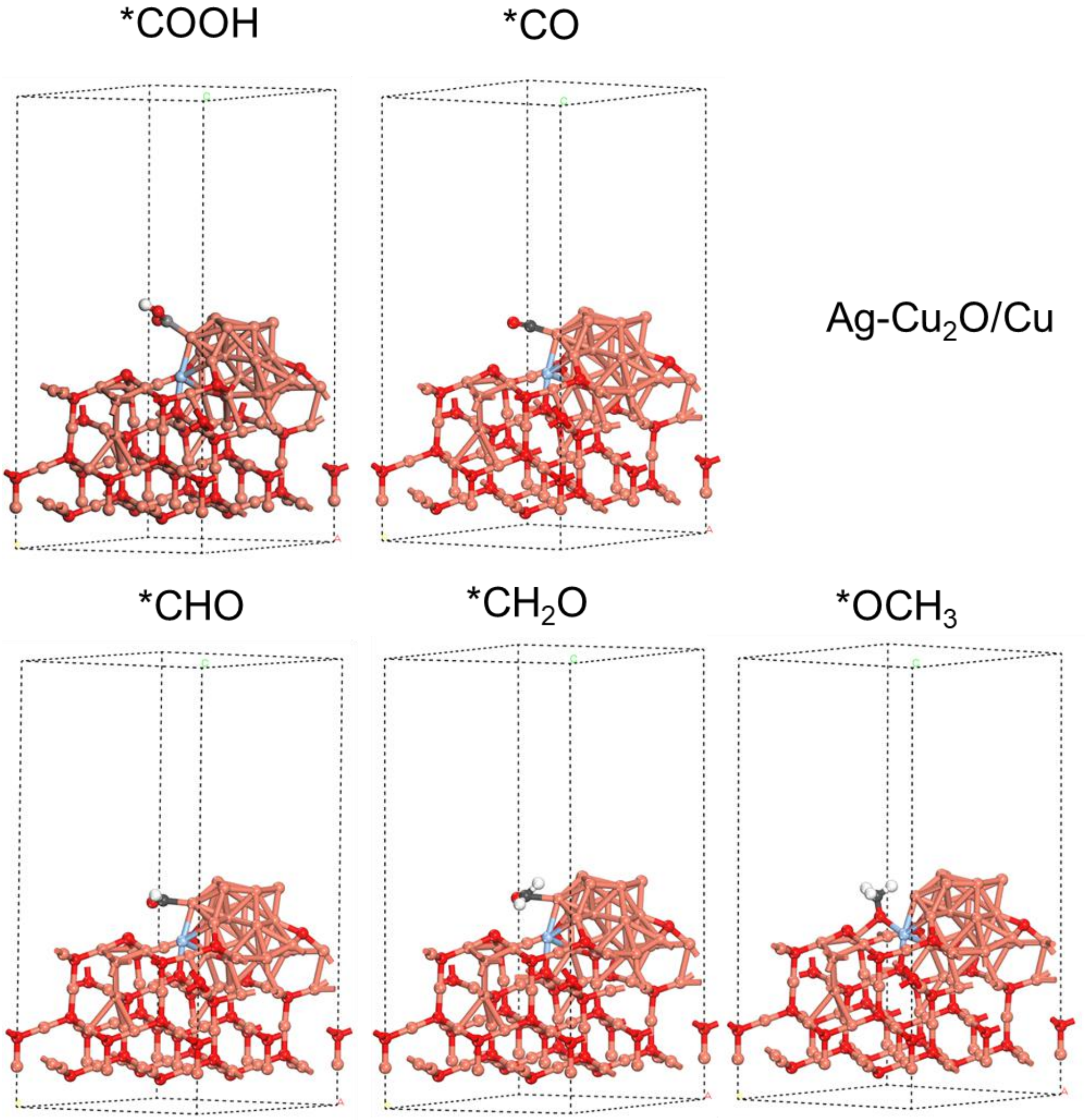

Supplementary Fig. 33. The optimized adsorption configurations of reaction intermediates on the Ag-Cu_2O/Cu structure. The atoms in orange, light blue, grey, red and white represent Cu, Ag, C, O and H, respectively.

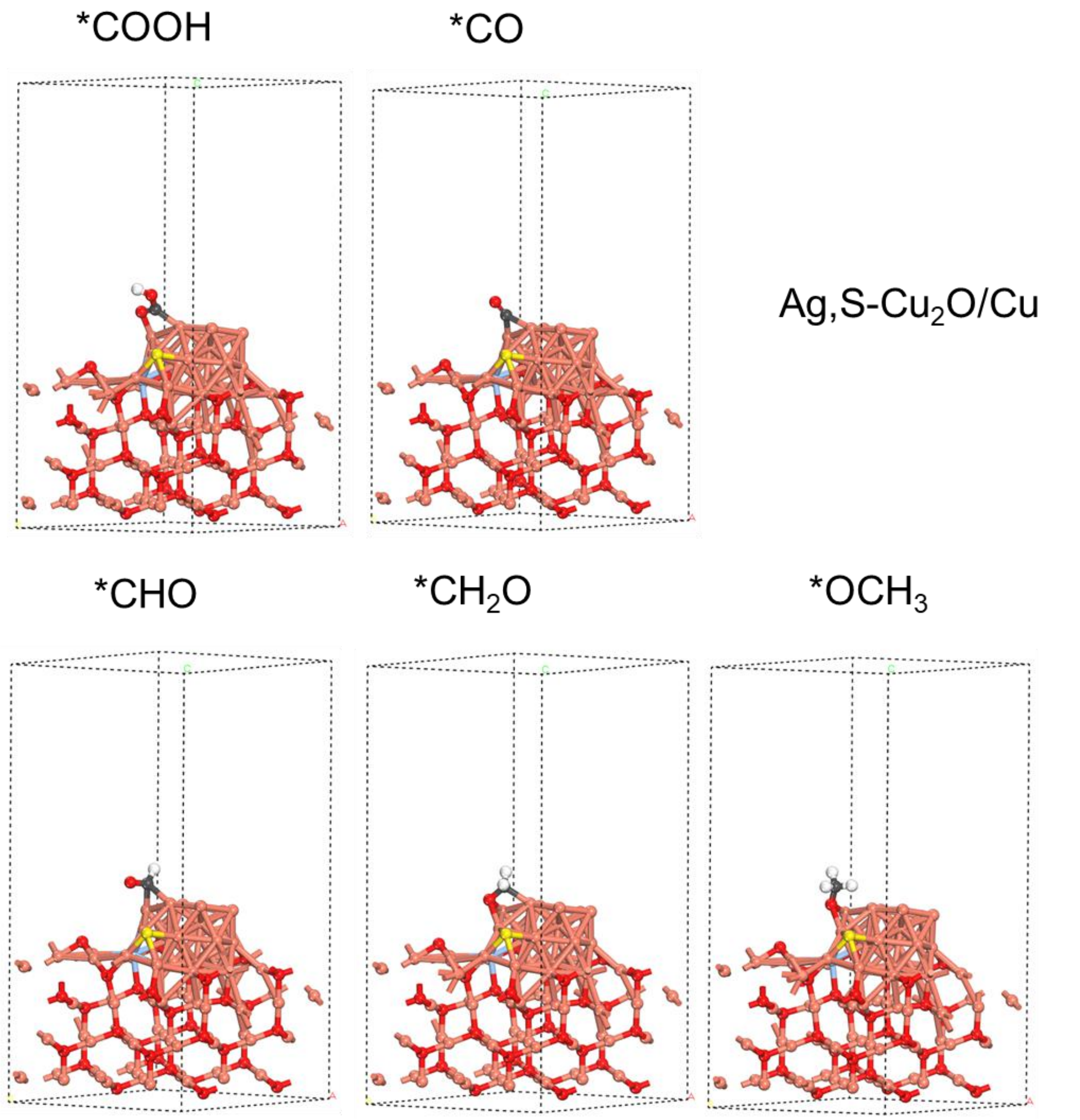

Supplementary Fig. 34. The optimized adsorption configurations of reaction intermediates on the Ag,S-Cu_2O/Cu structure. The atoms in orange, light blue, grey, red, yellow and white represent Cu, Ag, C, O, S and H, respectively.

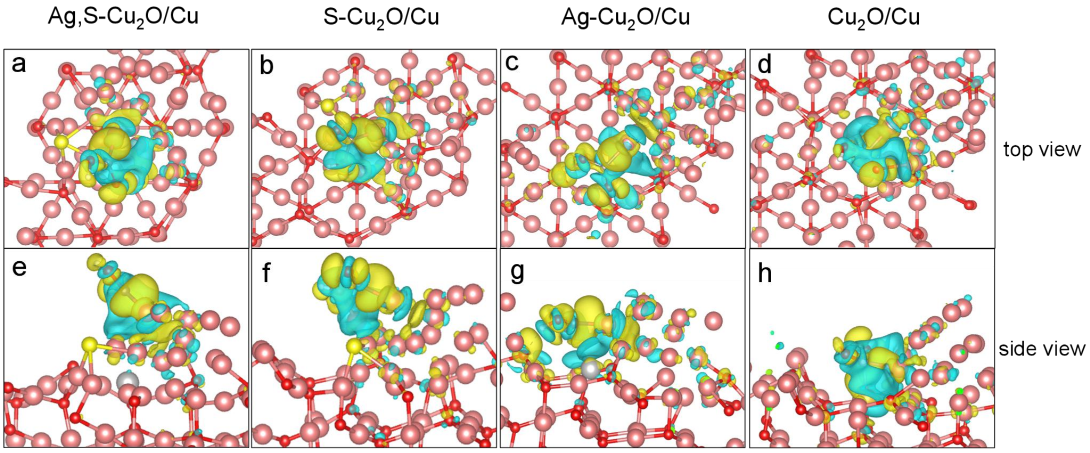

Supplementary Fig. 35. Differential charge densities of (a, e) Ag,S-Cu_2O/Cu, (b, f) S-Cu_2O/Cu, (c, g) Ag-Cu_2O/Cu and (d, h) Cu_2O/Cu with and without COOH adsorption in the top view and side view. Yellow and blue contours represent electron accumulation and depletion, respectively.

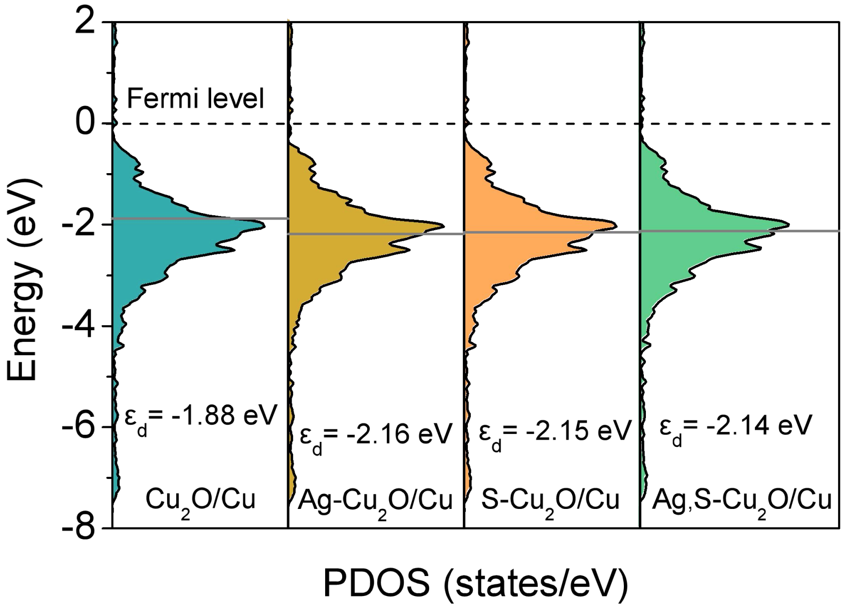

Supplementary Fig. 36. Projected density of state (PDOS) of the Cu atoms in Cu_2O/Cu, Ag-Cu_2O/Cu, S-Cu_2O/Cu and Ag,S-Cu_2O/Cu with the d-band center position marked by gray line and the Fermi level set as zero.

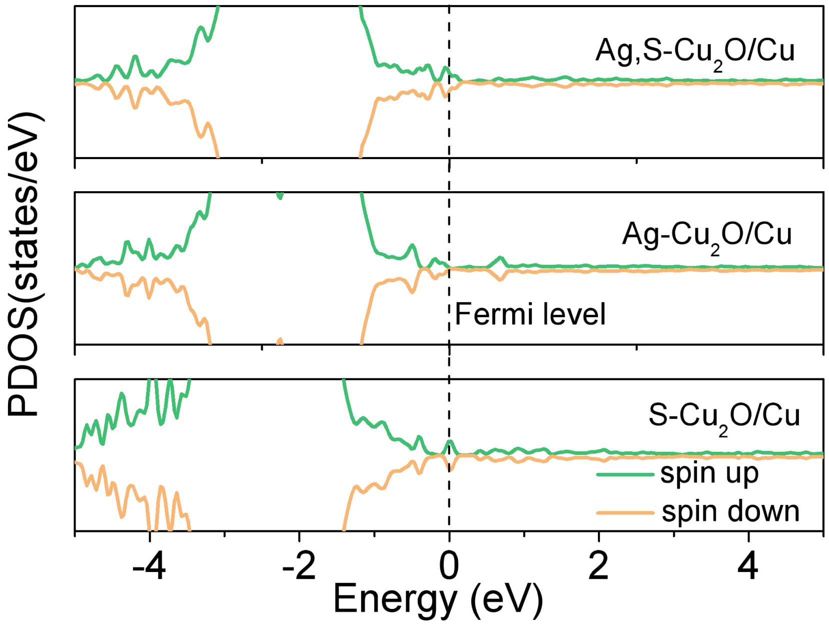

Supplementary Fig. 37. Projected density of state (PDOS) of Cu atom active center near the Fermi level.

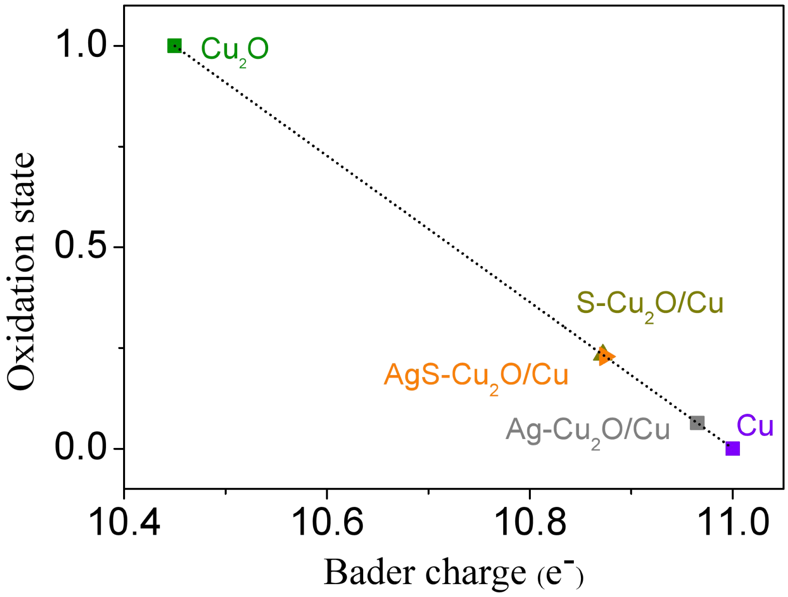

Supplementary Fig. 38. Oxidation states of interface Cu atoms obtained by Bader charge analysis in Ag-Cu_2O/Cu, S-Cu_2O/Cu and Ag,S-Cu_2O/Cu.

2.Supplementary Table

Supplementary Table 1. Faradaic efficiency (FE) of methanol and current density (*j*) of as-prepared Ag,S-Cu_2O/Cu in electrochemical CO_2 reduction comparing with some state of the art catalysts.

Electrocatalysts	Electrolyte	Potential (V)	$FE_{methanol}$ (%)	*j*(mA/cm^2)	References
Ag,S-Cu_2O/Cu	$BMImBF_4$/H_2O (1:3)	-1.18V vs RHE	67.4	122.6	This work
Pd/SnO_2	0.1 M $NaHCO_3$	−0.24 V vs RHE	54.8	1.5	S1
[PYD]@Pd[a]	0.5 M KCl	−0.6 V vs SCE	35	45	S2
Cu_2O/ZnO	0.5 M $KHCO_3$	−1.3 V vs Ag/AgCl	17.7	10.64	S3
Co/N-C	0.1M $NaHCO_3$	−0.90 V vs SCE	71.4	4	S4
Cu cluster-ZnO	0.1 M $KHCO_3$	−1.4 V vs Ag/AgCl	2.8	12	S5
$Co(CO_3)0.5(OH)$ $0.11H_2O$	0.1M $NaHCO_3$	−0.98 V vs SCE	97	0.6	S6
BP/CP[b]	0.1 M $KHCO_3$	−0.5 V vs RHE	92.0	0.2	S7
$Cu_{1.63}$ Se(1/3)	$BMImPF_6$(30 wt%)CH_3OH/ H_2O (5wt%)	-2.1V vs Ag/Ag^+	77.6	41.5	S8
$Pd_{83}Cu_{17}$	$BMImBF_4$(25 %)/H_2O(75%)	-2.1V vs Ag/Ag^+	80	31.8	S9
MoBi BMC[c] nanosheet	$BMImBF_4$/CH_3CN	-0.7V vs SHE	71.2	12.1	S10
Cu_2O/CuO-300	0.5 M $KHCO_3$	−1.3 V vs Ag/AgCl	6.46	46	S11
CoO/CN[d]/Ni	0.5 M $KHCO_3$	−0.7 V vs RHE	70.7	10.6	S12
CoPc/CNT[e]	0.1M $KHCO_3$	-0.94V vs RHE	44	10.6	S13
Pt_XZn/C	0.1M $KHCO_3$	-0.9V vs RHE	81.4	~0.4	S14
CuSAs/TCNFs[f]	0.1M $KHCO_3$	-0.9V vs RHE	44	93	S15
HKUST-1[g] MOF	0.5M $KHCO_3$	-0.9V vs Ag/AgCl	5.6	10.8	S16
BDD[h]	1.0M NH_3 aqueous solution	-1.3V vs Ag/AgCl	24.3	~0.2	S17
$Cu_{63.9}Au_{36.1}$/NCF[i]	0.5M $KHCO_3$	-1.1V vs SCE	15.9	/	S18
$CuBi_{12}$[j]	0.5M $KHCO_3$	-0.21V vs RHE	8.6	10	S19

Enzymes immobilized on carbon felt	0.05M phosphate buffer	-1.2V vs Ag/AgCl	10	~0.07	S20
Pt	10mM pyridoxine+0.1M KCl	-0.55V vs Ag/AgCl	5	/	S21
Pt	0.1M $LiClO_4$ in CH_3CN/H_2O (40% v/v)	-0.75 vs $Fc^{0/+}$	36	/	S22
Electrodeposited Cu_2O film	0.5M $NaHCO_3$	-1.1V vs SCE	38	/	S23

[a]PYD: pyridine derivative, [b]BP/CP: boron phosphide / carbon paper, [c]BMC: bimetallic chalcogenide, [d]CN: N-doped carbon, [e]CNT: carbon nanotube, [f]CuSAs/TCNFs: isolated Cu decorated through-hole carbon nanofibers, [g]HKUST-1:$[Cu_3(\mu_6 - C_9H_3O_6)_2]_n$, [h]BDD: boron-doped diamond, [i]NCF: nanoporous Cu film, [j]$CuBi_{12}$:Cu and Based MOF blends.

Supplementary Table 2. Elemental analysis of Ag,S-Cu_2O/Cu through ICP measurements

Element	Test value	Atom %
Cu	17.491 ppm	74.9
Ag	0.877 ppm	2.3
S	0.603 ppm	5.2

The catalyst was peeled off from the electrode. 2 mg catalyst (Ag,S-Cu_2O/Cu) was dissolved into HNO_3 solution (10 mL) with the help of ultrasound. Then, 1 mL of sample solution was diluted to 10 mL with deionized water, and measured with inductively coupled plasma (ICP, Thermo X Series II, Thermo Fisher Scientific). The rest should be the oxygen (17.6%). Therefore, the molar ratio of metallic Cu to Cu_2O in the Ag,S-Cu_2O/Cu catalyst can be roughly determined, which is about 1.1.

Supplementary Table 3. The correction of zero-point energy (ZPE), enthalpy effect, and entropy effect of the adsorbed and gaseous species. T = 298.15 K.

Cu_2O/Cu	ZPE/eV	U(T)/eV	H(T)/eV	G(T)/eV	S/eV·K^{-1}
*COOH	0.597	0.702	0.702	0.504	0.000663
*CO	0.188	0.260	0.260	0.119	0.000472
*CHO	0.444	0.503	0.503	0.389	0.000383
$*OCH_2$	0.772	0.842	0.842	0.726	0.000390
$*OCH_3$	1.091	1.188	1.188	1.001	0.000628

$S-Cu_2O/Cu$	ZPE/eV	U(T)/eV	H(T)/eV	G(T)/eV	S/eV·K^{-1}
*COOH	0.589	0.677	0.677	0.500	0.000594
*CO	0.183	0.265	0.265	0.090	0.000586
*CHO	0.428	0.496	0.496	0.360	0.000455
$*OCH_2$	0.714	0.790	0.790	0.627	0.000544
$*OCH_3$	1.060	1.140	1.140	0.989	0.000506

$Ag-Cu_2O/Cu$	ZPE/eV	U(T)/eV	H(T)/eV	G(T)/eV	S/eV·K^{-1}
*COOH	0.583	0.691	0.691	0.470	0.000743
*CO	0.184	0.241	0.241	0.124	0.000392
*CHO	0.434	0.500	0.500	0.369	0.000442
$*OCH_2$	0.715	0.775	0.775	0.655	0.000405
$*OCH_3$	1.037	1.118	1.118	0.963	0.000518

$Ag,S-Cu_2O/Cu$	ZPE/eV	U(T)/eV	H(T)/eV	G(T)/eV	S/eV·K^{-1}
*COOH	0.577	0.647	0.647	0.519	0.000427
*CO	0.183	0.242	0.242	0.122	0.000401
*CHO	0.435	0.507	0.507	0.349	0.000530
$*OCH_2$	0.695	0.748	0.748	0.634	0.000381
$*OCH_3$	1.092	1.190	1.190	0.998	0.000644

$Cd,S-Cu_2O/Cu$	ZPE/eV	U(T)/eV	H(T)/eV	G(T)/eV	S/eV·K^{-1}
*COOH	0.608	0.711	0.711	0.502	0.000701
*CO	0.179	0.257	0.257	0.096	0.000539
*CHO	0.467	0.539	0.539	0.403	0.000454
$*OCH_2$	0.761	0.843	0.843	0.690	0.000511
$*OCH_3$	1.100	1.197	1.197	1.005	0.000644

$Ag,I-Cu_2O/Cu$	ZPE/eV	U(T)/eV	H(T)/eV	G(T)/eV	S/eV·K^{-1}
*COOH	0.611	0.712	0.712	0.512	0.000670
*CO	0.188	0.268	0.268	0.096	0.000577

*CHO	0.411	0.478	0.478	0.337	0.000473
*OCH_2	0.750	0.824	0.824	0.676	0.000496
*OCH_3	1.068	1.153	1.153	0.977	0.000590

Au,S-Cu_2O/Cu	ZPE/eV	U(T)/eV	H(T)/eV	G(T)/eV	S/eV·K^{-1}
*COOH	0.607	0.712	0.712	0.504	0.000698
*CO	0.178	0.258	0.258	0.093	0.000552
*CHO	0.464	0.537	0.537	0.400	0.000460
*OCH_2	0.726	0.799	0.799	0.650	0.000500
*OCH_3	1.039	1.114	1.114	0.963	0.000507

Ag,Se-Cu_2O/Cu	ZPE/eV	U(T)/eV	H(T)/eV	G(T)/eV	S/eV·K^{-1}
*COOH	0.607	0.710	0.710	0.502	0.000698
*CO	0.193	0.270	0.270	0.100	0.000568
*CHO	0.447	0.513	0.513	0.378	0.000455
*OCH_2	0.757	0.830	0.830	0.682	0.000495
*OCH_3	1.053	1.155	1.155	0.944	0.000707

Zn,S-Cu_2O/Cu	ZPE/eV	U(T)/eV	H(T)/eV	G(T)/eV	S/eV·K^{-1}
*COOH	0.604	0.707	0.707	0.511	0.000658
*CO	0.176	0.255	0.255	0.093	0.000543
*CHO	0.438	0.500	0.500	0.365	0.000454
*OCH_2	0.731	0.800	0.800	0.666	0.000446
*OCH_3	1.041	1.116	1.116	0.970	0.000489

Supplementary Table 4. Bader charge of each atom before and after doping.

Atom	Bader charge (e^-)			
	Cu_2O/Cu	S-Cu_2O/Cu	Ag-Cu_2O/Cu	Ag,S-Cu_2O/Cu
1Cu	10.661	10.660	10.660	10.658
2Cu	10.501	10.480	10.519	10.512
3Cu	10.529	10.520	10.494	10.495
4Cu	10.720	10.572	10.670	10.566
5Cu	10.453	10.453	10.452	10.453
6Cu	10.471	10.474	10.475	10.466
7Cu	10.475	10.470	10.471	10.482
8Cu	10.450	10.661	10.458	10.765
9Cu	10.448	10.448	10.448	10.448
10Cu	10.466	10.471	10.472	10.467
11Cu	10.478	10.469	10.484	10.459
12Cu	10.450	10.696	10.485	10.649
13Cu	10.448	10.448	10.448	10.448
14Cu	10.470	10.474	10.474	10.469
15Cu	10.475	10.476	10.461	10.461
16Cu	10.452	10.467	10.444	10.501
17Cu	10.659	10.660	10.660	10.660
18Cu	10.502	10.524	10.504	10.501
19Cu	10.528	10.493	10.509	10.488
20Cu	10.722	10.790	10.781	10.816
21Cu	10.453	10.452	10.453	10.452
22Cu	10.471	10.472	10.476	10.466
23Cu	10.475	10.460	10.468	10.469
24Cu	10.451	10.448	10.465	10.481
25Cu	10.448	10.448	10.448	10.448
26Cu	10.468	10.471	10.472	10.466
27Cu	10.474	10.455	10.487	10.459
28Cu	10.502	10.570	10.518	10.499
29Cu	10.448	10.449	10.448	10.448
30Cu	10.469	10.472	10.475	10.457
31Cu	10.477	10.464	10.480	10.468
32Cu	10.501	10.957	10.518	10.906
33Cu	10.659	10.661	10.658	10.659
34Cu	10.532	10.508	10.519	10.512
35Cu	10.526	10.492	10.488	10.464
36Cu	10.722	10.774	10.784	10.756

37Cu	10.453	10.453	10.452	10.452
38Cu	10.471	10.475	10.466	10.474
39Cu	10.476	10.476	10.484	10.460
40Cu	10.426	10.448	10.437	10.467
41Cu	10.448	10.448	10.447	10.448
42Cu	10.468	10.475	10.467	10.472
43Cu	10.479	10.454	10.475	10.466
44Cu	10.436	10.440	10.443	10.453
45Cu	10.448	10.448	10.448	10.448
46Cu	10.470	10.475	10.475	10.474
47Cu	10.478	10.471	10.458	10.455
48Cu	10.451	10.441	10.438	10.434
49Cu	10.659	10.660	10.661	10.660
50Cu	10.501	10.518	10.498	10.497
51Cu	10.456	10.460	10.482	10.480
52Cu	10.727	10.728	10.452	10.453
53Cu	10.452	10.453	10.474	10.476
54Cu	10.470	10.474	10.484	10.492
55Cu	10.476	10.470	10.492	10.494
56Cu	10.498	10.510	10.448	10.447
57Cu	10.448	10.447	10.471	10.471
58Cu	10.467	10.473	10.492	10.486
59Cu	10.478	10.483	10.447	10.438
60Cu	10.453	10.447	10.448	10.448
61Cu	10.448	10.448	10.465	10.472
62Cu	10.469	10.476	10.485	10.487
63Cu	10.478	10.468	10.439	10.432
64Cu	10.432	10.446	10.888	10.967
65Cu	10.831	10.976	10.986	10.943
66Cu	11.030	10.936	11.025	10.989
67Cu	11.003	11.000	10.986	10.979
68Cu	11.010	11.007	11.056	11.055
69Cu	11.010	11.045	10.948	10.948
70Cu	11.003	10.957	11.034	10.816
71Cu	11.010	10.745	10.975	10.988
72Cu	11.009	11.006	10.965	10.874
73Cu	11.018	10.872	10.936	11.001
74Cu	11.008	11.019	11.036	11.026
75Cu	11.010	10.998	10.745	10.820
76Cu	10.778	10.855	10.945	10.854

77Cu	11.032	10.933	10.971	10.786
78Cu	11.029	10.776	10.800	10.998
79Cu	10.784	10.996	10.799	10.801
80Cu	10.775	10.775		
80Ag			10.922	10.883
81O	7.038	7.039	7.038	7.038
82O	7.035	7.021	7.042	7.033
83O	7.027	7.049	7.040	7.053
84O	7.044	7.007	7.032	7.006
85O	7.007	6.996	7.006	7.000
86O	6.989	7.033	6.991	7.015
87O	7.040	7.033	7.016	7.060
88O	7.024	7.039	7.048	7.039
89O	7.038	7.042	7.038	7.046
90O	7.035	7.037	7.020	7.045
91O	7.049	7.040	7.038	7.015
92O	7.045	7.006	7.012	7.006
93O	7.007	6.993	7.006	6.991
94O	6.998	7.016	6.993	7.039
95O	7.041	7.055	7.032	7.036
96O	7.025	7.039	7.034	7.039
97O	7.038	7.042	7.038	7.043
98O	7.034	7.032	7.046	7.036
99O	7.046	6.965	7.037	6.954
100O	6.992	7.006	6.967	7.007
101O	7.007	6.993	7.006	6.993
102O	6.998	7.035	7.000	7.032
103O	7.005	7.037	7.022	7.039
104O	7.024	7.038	7.028	7.038
105O	7.038	7.051	7.039	7.038
106O	7.034	7.041	7.033	7.012
107O	7.048	7.050	7.023	7.042
108O	7.047	7.007	7.043	7.007
109O	7.007	6.995	7.007	6.995
110O	6.998	7.013	6.992	7.039
111O	7.040	7.021	7.039	7.025
112O	7.011		7.024	
112S		6.614		6.600

3.References

S1. Zhang W, Qin Q, Dai L, Qin R, Zhao X, Chen X*, et al.* Electrochemical reduction of carbon dioxide to methanol on hierarchical Pd/SnO_2 nanosheets with abundant Pd–O–Sn interfaces. *Angew Chem Int Edit* **2018**, 57(30)**:** 9475-9479.

S2. Yang H-P, Qin S, Wang H, Lu J-X. Organically doped palladium: a highly efficient catalyst for electroreduction of CO_2 to methanol. *Green Chem* **2015**, 17(12)**:** 5144-5148.

S3. Albo J, Sáez A, Solla-Gullón J, Montiel V, Irabien A. Production of methanol from CO_2 electroreduction at Cu_2O and Cu_2O/ZnO-based electrodes in aqueous solution. *Appl Catal B Environ* **2015**, 176**:** 709-717.

S4. Huang J, Guo X, Yue G, Hu Q, Wang L. Boosting CH_3OH production in electrocatalytic CO_2 reduction over partially oxidized 5 nm cobalt nanoparticles dispersed on single-layer nitrogen-doped graphene. *ACS Appl Mater Interfaces* **2018**, 10(51)**:** 44403-44414.

S5. Andrews E, Ren M, Wang F, Zhang Z, Sprunger P, Kurtz R*, et al.* Electrochemical reduction of CO_2 at Cu nanocluster/(100) ZnO electrodes. *J Electrochemical Soc* **2013**, 160(11)**:** H841.

S6. Huang J, Hu Q, Guo X, Zeng Q, Wang L. Rethinking Co $(CO_3)_{0.5}(OH)\cdot 0.11H_2O$: a new property for highly selective electrochemical reduction of carbon dioxide to methanol in aqueous solution. *Green Chem* **2018**, 20(13)**:** 2967-2972.

S7. Mou S, Wu T, Xie J, Zhang Y, Ji L, Huang H*, et al.* Boron Phosphide Nanoparticles: A Nonmetal Catalyst for High - Selectivity Electrochemical Reduction of CO_2 to CH_3OH. *Adv Mater* **2019**, 31(36)**:** 1903499.

S8. Yang D, Zhu Q, Chen C, Liu H, Liu Z, Zhao Z*, et al.* Selective electroreduction of carbon dioxide to methanol on copper selenide nanocatalysts. *Nat Commun* **2019**, 10(1)**:** 677.

S9. Lu L, Sun X, Ma J, Yang D, Wu H, Zhang B*, et al.* Highly efficient electroreduction of CO_2 to methanol on palladium-copper bimetallic aerogels. *Angew Chem Int Edit* **2018**, 57(43)**:** 14149-14153.

S10. Sun X, Zhu Q, Kang X, Liu H, Qian Q, Zhang Z*, et al.* Molybdenum-bismuth bimetallic chalcogenide nanosheets for highly efficient electrocatalytic reduction of carbon dioxide to methanol. *Angew Chem Int Edit* **2016**, 128(23)**:**

6883-6887.

S11. Roy A, Jadhav HS, Gil Seo J. Cu_2O/CuO Electrocatalyst for Electrochemical Reduction of Carbon Dioxide to Methanol. *Electroanalysis* **2021**, 33(3)**:** 705-712.

S12. Wang L, Xu Y, Chen T, Wei D, Guo X, Peng L*, et al.* Ternary heterostructural CoO/CN/Ni catalyst for promoted CO_2 electroreduction to methanol. *J Catal* **2021**, 393**:** 83-91.

S13. Wu Y, Jiang Z, Lu X, Liang Y, Wang H. Domino electroreduction of CO_2 to methanol on a molecular catalyst. *Nature* **2019**, 575(7784)**:** 639-642.

S14. Payra S, Shenoy S, Chakraborty C, Tarafder K, Roy S. Structure-sensitive electrocatalytic reduction of CO_2 to methanol over carbon-supported intermetallic PtZn nano-alloys. *ACS Appl Mater Interfaces* **2020**, 12(17)**:** 19402-19414.

S15. Yang H, Wu Y, Li G, Lin Q, Hu Q, Zhang Q*, et al.* Scalable production of efficient single-atom copper decorated carbon membranes for CO_2 electroreduction to methanol. *J Am Chem Soc* **2019**, 141(32)**:** 12717-12723.

S16. Albo J, Vallejo D, Beobide G, Castillo O, Castaño P, Irabien A. Copper-based metal-Organic porous materials for CO_2 electrocatalytic reduction to alcohols. *ChemSusChem* **2017**, 10(6)**:** 1100-1109.

S17. Jiwanti PK, Natsui K, Nakata K, Einaga Y. Selective production of methanol by the electrochemical reduction of CO_2 on boron-doped diamond electrodes in aqueous ammonia solution. *RSC advances* **2016**, 6(104)**:** 102214-102217.

S18. Jia F, Yu X, Zhang L. Enhanced selectivity for the electrochemical reduction of CO_2 to alcohols in aqueous solution with nanostructured Cu-Au alloy as catalyst. *J Power Sources* **2014**, 252**:** 85-89.

S19. Albo J, Perfecto-Irigaray M, Beobide G, Irabien A. Cu/Bi metal-organic framework-based systems for an enhanced electrochemical transformation of CO_2 to alcohols. *J CO_2 Util* **2019**, 33**:** 157-165.

S20. Schlager S, Dumitru LM, Haberbauer M, Fuchsbauer A, Neugebauer H, Hiemetsberger D*, et al.* Electrochemical reduction of carbon dioxide to methanol by direct injection of electrons into immobilized enzymes on a modified electrode. *ChemSusChem* **2016**, 9(6)**:** 631-635.

S21. Lee JH, Lauw SJ, Webster RD. The electrochemical reduction of carbon dioxide (CO_2) to methanol in the presence of pyridoxine (vitamin B_6). *Electrochem Commun* **2016**, 64**:** 69-73.

S22. Giesbrecht PK, Herbert DE. Electrochemical reduction of carbon dioxide to methanol in the presence of benzannulated dihydropyridine additives. *ACS Energy Let* **2017**, 2(3)**:** 549-555.

S23. Le M, Ren M, Zhang Z, Sprunger PT, Kurtz RL, Flake JC. Electrochemical reduction of CO_2 to CH_3OH at copper oxide surfaces. *J Electrochem Soc* **2011**, 158(5)**:** E45.
